# Automating the Generation of Antimicrobial Resistance Surveillance Reports: Proof-of-Concept Study Involving Seven Hospitals in Seven Countries

**DOI:** 10.2196/19762

**Published:** 2020-10-02

**Authors:** Cherry Lim, Thyl Miliya, Vilada Chansamouth, Myint Thazin Aung, Abhilasha Karkey, Prapit Teparrukkul, Batra Rahul, Nguyen Phu Huong Lan, John Stelling, Paul Turner, Elizabeth Ashley, H Rogier van Doorn, Htet Naing Lin, Clare Ling, Soawapak Hinjoy, Sopon Iamsirithaworn, Susanna Dunachie, Tri Wangrangsimakul, Viriya Hantrakun, William Schilling, Lam Minh Yen, Le Van Tan, Htay Htay Hlaing, Mayfong Mayxay, Manivanh Vongsouvath, Buddha Basnyat, Jonathan Edgeworth, Sharon J Peacock, Guy Thwaites, Nicholas PJ Day, Ben S Cooper, Direk Limmathurotsakul

**Affiliations:** 1 Mahidol Oxford Tropical Medicine Research Unit Faculty of Tropical Medicine Mahidol University Bangkok Thailand; 2 Nuffield Department of Medicine Centre for Tropical Medicine and Global Health University of Oxford Oxford United Kingdom; 3 Cambodia-Oxford Medical Research Unit Angkor Hospital for Children Siem Reap Cambodia; 4 Lao-Oxford-Mahosot Hospital-Wellcome Trust Research Unit Mahosot Hospital Vientiane Lao People's Democratic Republic; 5 North Okkalapa General and Teaching Hospital Yangon Myanmar; 6 Patan Hospital Kathmandu Nepal; 7 Oxford University Clinical Research Unit Patan Hospital Kathmandu Nepal; 8 Sunpasitthiprasong Hospital Ubon Ratchathani Thailand; 9 Department of Infectious Diseases Centre for Clinical Infection and Diagnostic Research King's College London & Guy's and St Thomas' NHS Foundation Trust London United Kingdom; 10 Hospital for Tropical Diseases Ho Chi Minh City Vietnam; 11 Brigham and Women's Hospital and Harvard Medical School Boston, MA United States; 12 Myanmar Oxford Clinical Research Unit Yangon Myanmar; 13 Oxford University Clinical Research Unit Ho Chi Minh City Vietnam; 14 Shoklo Malaria Research Unit and Mahidol Oxford Tropical Medicine Research Unit Faculty of Tropical Medicine Mahidol University Mae Sot Thailand; 15 Department of Disease Control Bureau of Epidemiology Ministry of Public Health Nonthaburi Thailand; 16 Department of Disease Control Office of International Cooperation Ministry of Public Health Nonthaburi Thailand; 17 Division of Communicable Diseases Department of Disease Control Ministry of Public Health Nonthaburi Thailand; 18 Institute of Research and Education Development University of Health Sciences Vientiane Lao People's Democratic Republic; 19 Department of Medicine University of Cambridge Cambridge United Kingdom

**Keywords:** antimicrobial resistance, surveillance, report, data analysis, application

## Abstract

**Background:**

Reporting cumulative antimicrobial susceptibility testing data on a regular basis is crucial to inform antimicrobial resistance (AMR) action plans at local, national, and global levels. However, analyzing data and generating a report are time consuming and often require trained personnel.

**Objective:**

This study aimed to develop and test an application that can support a local hospital to analyze routinely collected electronic data independently and generate AMR surveillance reports rapidly.

**Methods:**

An offline application to generate standardized AMR surveillance reports from routinely available microbiology and hospital data files was written in the R programming language (R Project for Statistical Computing). The application can be run by double clicking on the application file without any further user input. The data analysis procedure and report content were developed based on the recommendations of the World Health Organization Global Antimicrobial Resistance Surveillance System (WHO GLASS). The application was tested on Microsoft Windows 10 and 7 using open access example data sets. We then independently tested the application in seven hospitals in Cambodia, Lao People’s Democratic Republic, Myanmar, Nepal, Thailand, the United Kingdom, and Vietnam.

**Results:**

We developed the AutoMated tool for Antimicrobial resistance Surveillance System (AMASS), which can support clinical microbiology laboratories to analyze their microbiology and hospital data files (in CSV or Excel format) onsite and promptly generate AMR surveillance reports (in PDF and CSV formats). The data files could be those exported from WHONET or other laboratory information systems. The automatically generated reports contain only summary data without patient identifiers. The AMASS application is downloadable from https://www.amass.website/. The participating hospitals tested the application and deposited their AMR surveillance reports in an open access data repository.

**Conclusions:**

The AMASS is a useful tool to support the generation and sharing of AMR surveillance reports.

## Introduction

Generating and sharing antimicrobial resistance (AMR) surveillance reports are fundamental elements of actions against AMR infections at local, national, and international levels. Information on patterns of antimicrobial susceptibility is important to guide empiric choice of therapy, monitor resistance trends, and detect outbreaks of AMR infections at the local level [1-3]. Combining data and reports at the national level provides evidence to inform the implementation of national action plans, decide on resource allocation for interventions, and monitor the impact of those interventions [3-5]. The Review on AMR chaired by Jim O’Neill estimated that 700,000 global deaths are attributable to AMR infections each year (including bacterial infections and tuberculosis) [1,6], and they have an enormous global impact [7,8]. While this represents a very rough estimate subject to well-documented limitations [1,9,10], the report importantly highlighted the need for improved AMR surveillance.

Methods to analyze data and generate AMR surveillance reports are gradually being standardized worldwide [3,11-15]. Recently, the World Health Organization (WHO) launched the Global Antimicrobial Resistance Surveillance System (GLASS) with a defined protocol for AMR surveillance data collection for certain high-priority pathogens and resistance phenotypes [2,11,16]. In general, it is recommended that (1) repeat isolates of a given bacterial species from individual patients should be removed from the calculations and (2) data should be stratified by origin of infection (community or hospital) whenever possible [11,15]. A simple deduplication process is to include only the first isolate of a species per patient per specimen type per survey period in the report [3,11]. The origin of infection is defined by using specimen collection date, location type (inpatient or outpatient setting), and hospital admission date for inpatient isolates as a proxy to define where the infection was likely contracted (community or hospital) [3,11].

Even in areas where AMR surveillance data are available, there are many barriers to utilizing such data [17]. Many hospitals in low and middle-income countries (LMICs) lack the time and resources needed to analyze the data (particularly to deduplicate and validate the accuracy of the summary data), write reports, and release the data or reports [17]. There are open-access laboratory information systems (LISs) [18] and microbiology laboratory database software, including WHONET [19], that are useful for recording and analyzing the data, and generating figures to support the generation of AMR surveillance reports for hospitals in LMICs. Most of these systems have limited ability to generate summary reports for immediate use and be operated by nontechnical staff. In addition, to generate AMR surveillance reports stratified by the origin of infection, additional data on hospital admission are frequently needed [20-23]. This is because hospital admission dates are not generally collected in microbiology laboratory data files. In many hospital settings, microbiology and hospital admission data are held in separate computers or systems with restricted access. Even in high-income countries, many hospitals lack well-trained clinical microbiologists, epidemiologists, or data experts with adequate skills in statistical software (such as R, SAS, SPSS, and STATA) to merge and deduplicate data in the separated databases and generate reports stratified by the origin of infection.

Here, we developed an application termed the AutoMated tool for Antimicrobial resistance Surveillance System (AMASS), which can support a local hospital to independently analyze routinely collected electronic data and rapidly generate AMR surveillance reports. We tested the AMASS in seven hospitals in Cambodia, Lao People’s Democratic Republic, Myanmar, Nepal, Thailand, the United Kingdom, and Vietnam, and deposited the report from each hospital in an open-access platform.

## Methods

### Design of the Application

The tool operates by reading and processing the raw data files to automatically produce AMR surveillance reports. The application was designed to be open access, user friendly, and highly compatible with readily available data sets at local hospitals, and have high data security. To ensure the tool is fully open access and can run in a standalone inexpensive computer even without internet access, we built the application in R (version 3.6.2; R Project for Statistical Computing), which is a free software environment. We then gave the application a user-friendly interface, which only requires double clicking on the application file to run the automation without the need to understand the R program. We decided to include both R portable (version 3.4.3; R Project for Statistical Computing) and RStudio (version 1.1.423; RStudio, Inc) within the downloadable package so that the application can run without the need to install R or any program prior to running the application. We designed the application so that it reads raw data files in either CSV or Excel format as many hospitals in LMICs can export their data in one of these formats [17]. Hospitals that store microbiology data and hospital data in software, such as WHONET [19], can export data in CSV or Excel format. During the development process, we revised the R codes repeatedly to ensure that the application would produce easy-to-use and easy-to-share outputs. Table 1 documents the features that we focused on when designing the application [24,25].

**Table 1:** Features of the AutoMated tool for Antimicrobial resistance Surveillance System (AMASS).

Feature	Description
Open access	The AutoMated tool for Antimicrobial resistance Surveillance System (AMASS) is open access and can be downloaded [24,25]. The AMASS was developed using R, which is a free software. In the download package for the AMASS (AMASS.zip), there is a folder that contains R-portable and RStudio, which support data processing and analysis to generate the antimicrobial resistance (AMR) surveillance report automatically. The AMASS is under CC-BY 4.0 license. Users can share (copy and redistribute the material in any medium or format) and modify the R codes of the AMASS under the terms and conditions of the Creative Commons license.
User friendly	The AMASS can be run by double clicking on the application icon. Data analysis and AMR surveillance report generation are automated by the AMASS application. Data cleaning, deduplication, and analysis are performed rapidly (it takes about 1-3 minutes to automatically produce an AMR surveillance report using example data sets provided in the AMASS package). No additional program or software is needed. All the essential software is stored in the AMASS package and will operate automatically after double clicking the application file (AMASS.bat). Users do not need to understand R program or write any codes to run the AMASS application.
Highly compatible	The AMASS works with raw data files in either CSV or Excel format, which can be commonly exported from WHONET and other software, programs, or data management systems used for microbiology data and hospital admission data. The AMASS uses data dictionary files (in Excel format) to accommodate data exported from different software, programs, or systems that may have different ways to name data variables and data values. The AMASS dictionary files can be reused by users in the future (eg, monthly, quarterly, and yearly) if the structures of the new raw microbiology data file and hospital admission data file remain unchanged. The AMASS uses a tier-based approach based on availability of raw data files to generate reports. Users with limited data availability (eg, microbiology data with only culture positive results) can still utilize the deduplication and report generation functions of the AMASS. Users with additional data (eg, microbiology data with culture negative results and hospital admission date data) will receive additional reports (eg, sample-based surveillance reports with stratification by infection origin).
High data security	The AMASS does not require the internet for operation. Users do not have to transfer raw individual data (which may contain identifiable information) to any institution outside of the hospital to analyze the data and generate the reports. The AMASS can be run on a standalone computer within the local hospital under local data security. Hence, the AMASS does not increase any risks of breaching individual patient data confidentiality.
Easy-to-use outputs	The automatically generated AMR surveillance report is in PDF format, which is easy to print, read, and share within and outside the hospital.
Easy-to-share outputs	The report (in PDF format) and aggregated summary data files (in CSV format) contain no individual-level patient data and can be readily shared with national and international organizations.

A schematic overview of input data, data processing, statistical analysis, and output is shown in Multimedia Appendix 1. In brief, input data are microbiology data files (in CSV or Excel format) with or without hospital data files, which can be exported from WHONET or a LIS with data export capacity. The data processing and analysis algorithms, including data deduplication, were developed based on the WHO GLASS recommendations [2,11]. The reports were designed to have a format similar to that of the WHO GLASS 2018 report [14].

The application deduplicates the data by including only the first isolate per sample type per pathogen per survey period for each patient [11]. The application currently includes only blood specimens and the following eight pathogens: *Acinetobacter* spp., *Escherichia coli*, *Enterococcus* spp., *Klebsiella* *pneumoniae*, *Salmonella* spp., *Staphylococcus aureus*, *Streptococcus* *pneumoniae*, and *Pseudomonas* *aeruginosa*. Both *Enterococcus* spp. and *P. aeruginosa* were added on top of the six priority pathogens described by the WHO GLASS for bacteremia [2,26] because both pathogens are common causes of bacteremia [15,20], and were included in the 2015 global priority list of AMR bacteria [26]. The list of pathogen-antibiotic combinations was modified from the WHO GLASS (Multimedia Appendix 2). Infections are stratified into community-origin or hospital-origin infections using hospital admission dates and specimen collection dates, when available [2,11]. Patients with the first specimen culture positive for the pathogen taken in the outpatient setting or on the first or second day of hospitalization are classified as having community-origin infection [2,11]. Patients with the first specimen culture positive for the pathogen taken on hospital day three or later are classified as having hospital-origin infection [2,11]. Alternatively, in cases where users have data on the origin of infection assigned by the attending physician or infection control team of the hospital, the AMASS can instead use those categorizations to stratify the infection into community-origin or hospital-origin infection. Prevalence and incidence rates are estimated based on the recommendations of the WHO GLASS (Multimedia Appendix 3) [2,11]. An additional report on mortality involving AMR and non-AMR infections is generated when mortality data are available in the hospital admission data file. The term “mortality involving AMR and antimicrobial-susceptible infections” is used because the mortality reported in the hospital admission data represents mortality from any cause, not necessarily attributable to AMR [1]. This measure of mortality includes deaths caused or contributed by other underlying and intermediate causes. Therefore, the term “involving AMR infections” is used, in accordance with the term used by the UK Office for National Statistics [27,28]. The AMASS used the Wilson method to estimate the confidence intervals for proportions.

### Example Data Sets

Two example data sets are provided in the downloadable package of the AMASS application. The first example data set is the open-access demonstration file from WHONET [19]. The second example data set is a synthetic data set generated for the AMASS. The second example data set was created to represent a large data set from a 1000-bed hospital, containing both microbiology and hospital admission data. A detailed description on how the second example data set was generated is provided in Multimedia Appendix 4. In brief, two synthetic data files (microbiology_data.xls and hospital_admission_data.xls files) were generated based on the summary data in the AMR surveillance report from 2015 of Sunpasitthiprasong Hospital, Ubon Ratchathani, Thailand. Variables in the microbiology data file include hospital number, specimen type, specimen collection date, culture result, and antibiotic susceptibility testing result, and each row contains information for each specimen. Variables in the hospital admission data file include hospital number, age, sex, admission date, discharge date, and in-hospital discharge outcome. All data were randomly generated using STATA 15.1 (StataCorp).

### Testing the AMASS Using Hospital Data

The following seven hospitals participated in the study: Angkor Hospital for Children in Cambodia, Mahosot Hospital in Lao People’s Democratic Republic, North Okkalapa General and Teaching Hospital in Myanmar, Patan Hospital in Nepal, Sunpasitthiprasong Hospital in Thailand, St Thomas’ Hospital in the United Kingdom, and Hospital for Tropical Diseases in Vietnam. The hospitals were selected because microbiology data are collected routinely, and they have prior experience in data quality controls. Moreover, the hospitals varied in the LIS used for data storage and are good examples to demonstrate the practicality and usability of the AMASS in different settings, to a wider audience. The study was approved by the Oxford Tropical Research Ethics Committee, University of Oxford, and local Ethics Committees. CL corresponded with the participating hospitals and demonstrated how the application operates using the example data files. The local hospital staff operated the application using their local data by themselves. All microbiology and hospital data were stored independently within their hospital computers under their local data protection standards. Automatically generated reports and anonymous summary data from each hospital are publicly available and deposited in data repositories with permission from each hospital.

## Results

### Overview of the AMASS

**Figure 1:**
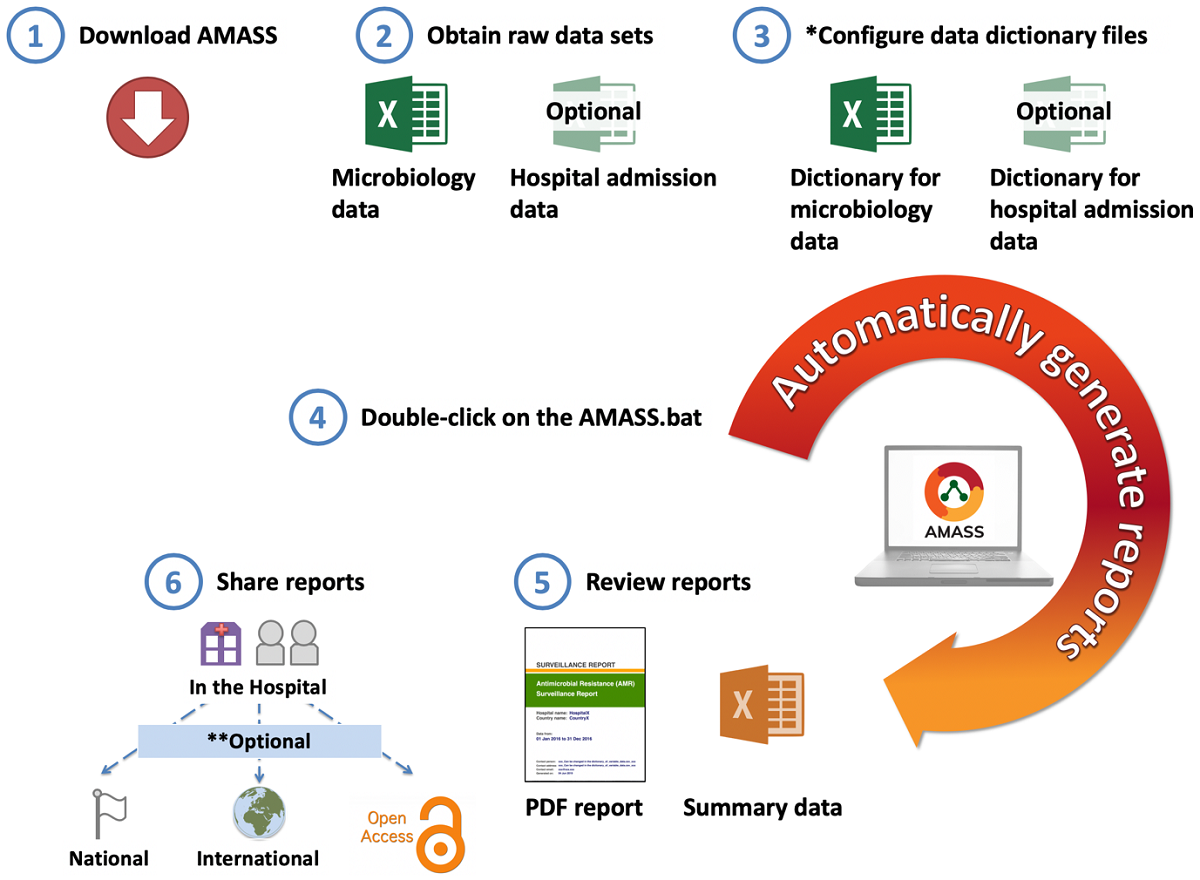
Conceptual flow of the AutoMated tool for Antimicrobial resistance Surveillance System (AMASS). Step 1 (download the AMASS) and step 3 (configure data dictionary files) are one-time steps. Step 2 (obtain data), step 4 (run the AMASS), step 5 (review report), and step 6 (share report) are ongoing steps that users could repeat regularly (ie, monthly or quarterly). *Two data dictionary files (in Excel format) are provided to allow the application to understand how variables and values of each variable are named in the raw data files in different settings. Those data dictionary files can be reused in the subsequent runs of the AMASS, as long as how variables and values of each variable are named in the raw data files remain the same. Details on how to configure the data dictionary can be found in Figure 2 and Multimedia Appendix 6. **The antimicrobial resistance (AMR) surveillance report and summary data generated contain no patient identifiable information. The decision to share the report and summary data to national or international AMR organizations is solely up to the jurisdiction of the hospital.

The AMASS application has been developed to support clinical microbiology laboratories to automatically analyze hospital local data files (in CSV or Excel format) and generate AMR surveillance reports (in PDF and CSV formats) promptly. Six steps are followed to generate the AMR surveillance reports (Figure 1 and Multimedia Appendix 5). First, download the AMASS package from the website [24]. Second, obtain the routinely collected raw microbiology data file and, if available, hospital admission data file, and then, save the data files in the folder of the AMASS application. Third, configure data dictionaries. Two data dictionaries are provided to accommodate different ways of naming variables (eg, sex and gender) and data values (eg, M and F, or male and female). The functioning of the data dictionaries is described in more detail in the “Illustration on How to Use the AMASS” section. Fourth, double click on the AMASS.bat file to run the application. Fifth, review and validate the AMR surveillance reports (generated in PDF format) and the anonymous summary data (generated in CSV format). Sixth, share the reports within the hospital, especially with the local infection control team. The reports and anonymous summary data contain no patient identifiers. Therefore, users may share the reports and anonymous summary data with national and international organizations or make the reports and anonymous summary data open access. The key features of the AMASS are listed in Table 1.

### Illustration on How to Use the AMASS

#### Input Requirements

To align with formats of commonly exported microbiology data and hospital admission data, the AMASS reads data files in both CSV and Excel formats. The microbiology data need to be in a wide format, meaning that each row should contain data from a single clinical isolate. The key variables required for the microbiology data file include patient identifier, specimen collection date, specimen type, culture result, and antimicrobial susceptibility test interpretation per antibiotic. The hospital admission data file is optional but, if available, also needs to be in a wide format. The key variables needed for the hospital admission data file include patient identifier, admission date, gender, age, and, if possible, in-hospital discharge outcome. The current version of the AMASS uses the Gregorian calendar and requires the date to be in the order of day, month, and year in any format (ie, either text [English] or numeric).

**Figure 2:**
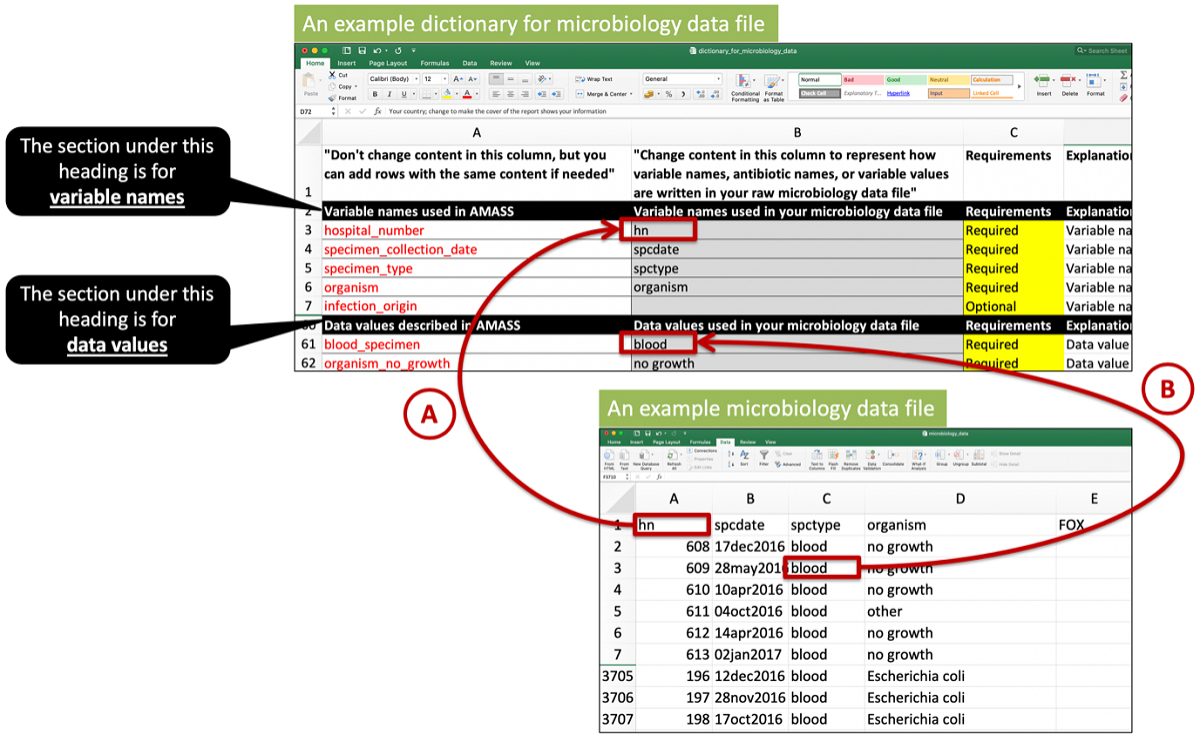
An example of how to complete a data dictionary file. For a first-time user, the user may need to complete a data dictionary file by filling in variable names used in the raw data files into the data dictionary files (eg, arrow A). This is to allow the AutoMated tool for Antimicrobial resistance Surveillance System (AMASS) to understand that the variable “hospital_number” used by the AMASS is named as “hn” in the user’s raw microbiology data file. Thereafter, users need to enter how data values are named in their raw data files (e.g. arrow B). This is to allow the AMASS to understand that the data value named “blood_specimen” is named as “blood” in user’s raw microbiology data file. Please note that the contents in the first column of the data dictionary file must remain unchanged. Users can add new rows but the content in the cell in the first column must not be changed. For example, users can define that both “*E. coli* (ESBL-producing strain)” and “*Escherichia coli*” in their raw microbiology data file mean “organism_escherichia_coli” by the AMASS. The example data dictionary files shown in the figure are available in the Example_Dataset_2 folder (within the AMASS download package).

There are two data dictionary files provided for the users to accommodate different ways of naming data variables and data values (Figure 2). The first data dictionary file (dictionary_for_microbiology_data.xls) is for the microbiology data file. For example, the AMASS uses the variable name “hospital_number” as a patient identifier (Row 3, Column A of the data dictionary file). In cases where the raw microbiology data file uses a different name for the patient identifier (eg. hn), users would need to fill “hn” in the data dictionary file (Row 3, Column B of the data dictionary file). This allows the AMASS application to know that the variable “hn” of the raw microbiology data file is the patient identifier (ie, “hospital_number”). The second data dictionary file (dictionary_for_hospital_admission_data.xls) is for the hospital admission data file, which is to be used likewise. Multimedia Appendix 6 presents a step-by-step tutorial on how to use and configure the data dictionaries.

#### Outputs Generated by the AMASS

The AMR surveillance reports generated from the AMASS are illustrated using the two open-access example data sets provided within the download package. Multimedia Appendicies 7 and 8 contain AMR surveillance reports generated from the first and second example data sets, respectively. Multimedia Appendix 5 illustrates how to test the AMASS using example data sets in a step-by-step fashion.

**Figure 3:**
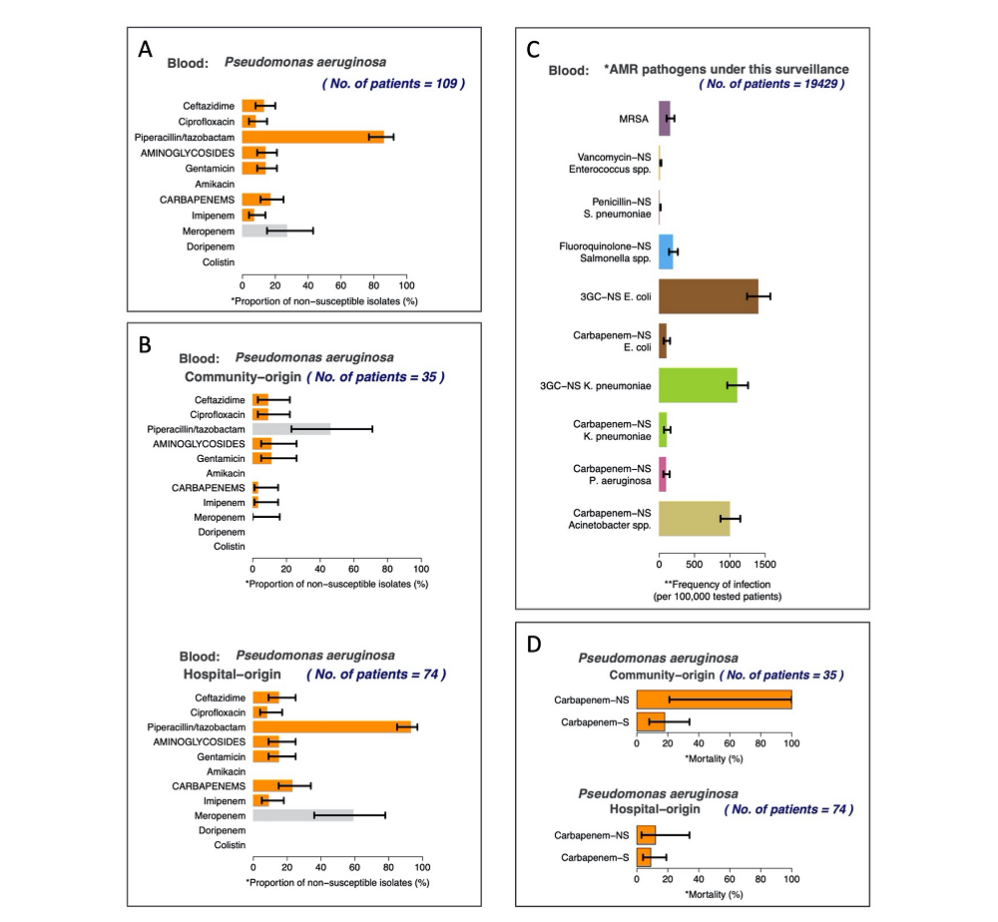
Examples of figures automatically generated by the AutoMated tool for Antimicrobial resistance Surveillance System (AMASS). All figures are from the report (Multimedia Appendix 8 Multimedia Appendix 8) automatically generated by the AMASS application using an example data set provided in the download package. Figure 3A represents the overall proportion of nonsusceptible (intermediate and resistant) isolates in an isolate-based report (section two in the report). Figure 3B represents the proportion of nonsusceptible isolates stratified by the origin of infection (section three in the report). Figure 3C represents the frequency of bloodstream infections per 100,000 tested patients (section four in the report). Figure 3D represents mortality involving antimicrobial-resistant and antimicrobial-susceptible bloodstream infections (section six in the report).

In short, the automatically generated report on AMR surveillance contains the following six sections (Figure 3): (1) data overview; (2) an isolate-based report; (3) an isolate-based report with stratification by origin of infection; (4) a sample-based report; (5) a sample-based report without stratification by infection; and (6) mortality involving AMR and antimicrobial-susceptible infections.

The AMASS uses a tier-based approach. When only the microbiology data file with the results of culture positive samples is available, only section one and two are automatically generated for users (as shown in Multimedia Appendix 7, generated from the first example data set). Section three is generated only when data on admission dates are available. This is because these data are required for stratification by the origin of infection. Section four is generated only when data of culture negative specimens (no microbial growth) are available in the microbiology data file. This is because these data are required for the sample-based approach. Section five is section four stratified by the origin of infection and is generated if admission date data are also available. Section six is generated only when mortality data are available (as shown in Multimedia Appendix 8, generated from the second example data set).

The AMASS also generates two log files. The first log file (generated in PDF format) is for users to validate the input data used by the AMASS to generate the AMR surveillance report. It contains information such as the total number of records analyzed, age distribution, number of missing values, and total number of isolates per organism in the raw microbiology data file. The second log file (generated in plain text format) could be used for consultation with R users, statisticians, or the AMASS development team in case of any technical issue when running the AMASS.

### Testing the AMASS Using Hospital Data

**Figure 4:**
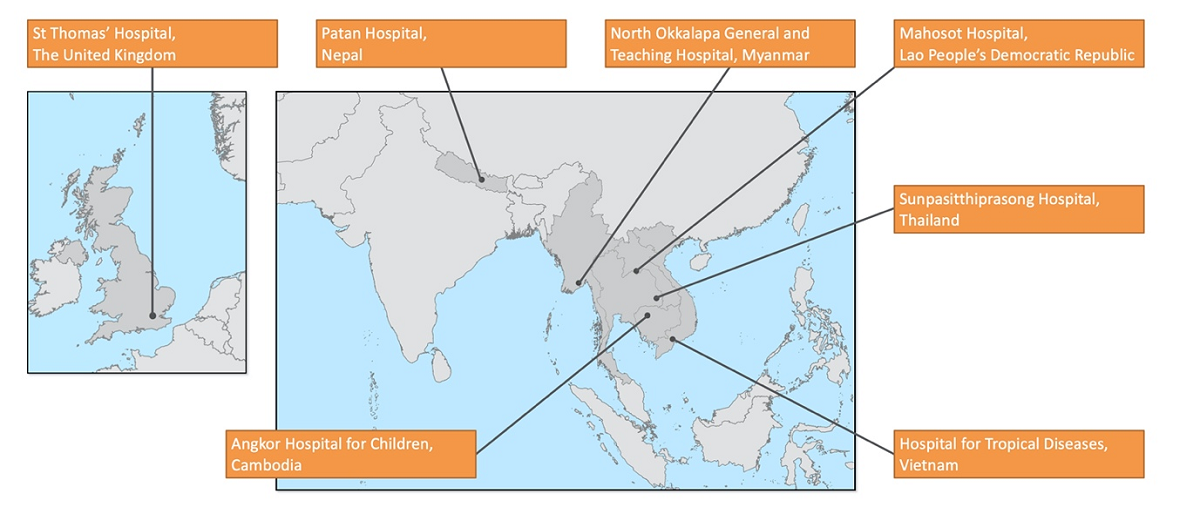
A map of participating hospitals and examples of summary data from the automatically generated antimicrobial resistance surveillance reports. The reports and summary data from St Thomas’ Hospital, Patan Hospital, North Okkalapa General and Teaching Hospital, Mahosot Hospital, Sunpasitthiprasong Hospital, Hospital for Tropical Diseases, and Angkor Hospital for Children are open access [28-34].

The AMASS was tested in seven hospitals in seven countries (Figure 4). The hospitals varied in data availability, data structure, naming of the variables, and definitions for data values (Multimedia Appendix 9). Overall, the proportions of patients having *Escherichia coli* bacteremia caused by third-generation cephalosporin-resistant isolates ranged from 19% to 85%. The incidence rates of *Escherichia coli* bacteremia caused by third-generation cephalosporin-resistant isolates ranged from 283 to 2737 per 100,000 tested patients among participating hospitals with available data on negative cultures.

All participating hospitals deposited their output files and their data dictionary files at figshare [29-35]. Angkor Hospital for Children, North Okkalapa General and Teaching Hospital, and Sunpasitthiprasong Hospital had all six sections of the AMR surveillance report, as microbiology and hospital admission data, data on negative cultures, and in-hospital discharge outcome data were available. Mahosot Hospital and Patan Hospital had sections one and two of the AMR surveillance report, as only microbiology data on positive cultures were available to test in the AMASS. St Thomas’ Hospital had sections one, two, three, and six of the AMR surveillance report because negative culture data were not available to test in the AMASS. Hospital for Tropical Diseases had sections one, two, and four available, as hospital admission data were not available to test in the AMASS.

Data storage systems varied across the hospitals. Angkor Hospital for Children used the ACORN LIS that is based on the Microsoft Access program [36]. Mahosot Hospital used a local LIS that is also based on the Microsoft Access program [37]. Sunpasitthiprasong Hospital used the MLAB program [38]. St Thomas’ Hospital used ICNET [39], which is a commercial clinical surveillance software package. North Okkalapa General and Teaching Hospital used WHONET 2019 (modernized version of WHONET 5.6) [19]. Patan Hospital and Hospital for Tropical Diseases used an in-house LIS based on the MySQL system. Inputting data dictionary files for the first time took about 1 to 3 hours. However, the data dictionary files could be stored and reused when the raw microbiology data file or hospital admission data file was revised or updated. North Okkalapa General and Teaching Hospital used the data dictionary file from the example data set (in the Example_Dataset_1_WHONET folder), which was generated to comply with WHONET exported data (in .XLSX format; Multimedia Appendix 10 illustrates how microbiology data were exported from WHONET 5.6). This saved the time and effort needed to complete the data dictionaries for the hospital.

We found that the AMASS took about 1 to 3 minutes to run and automatically generate an AMR surveillance report using local data and local hospital computers. The AMASS works on Microsoft Windows 10 and with data containing a non-English (Latin and non-Latin characters) language. For example, “blood_specimen” was recorded as “
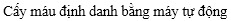
” in the raw microbiology excel file at Hospital for Tropical Diseases, Vietnam. As another example, “male” was recorded as “
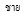
” and “female” was recorded as “
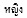
” in the raw hospital admission excel file at Sunpasitthiprasong Hospital, Thailand. By completing data dictionary files, users could use the AMASS to analyze the data and generate the AMR surveillance report even though the raw data were recorded in a local language.

All participating hospitals had previous experience in data verification and in AMR surveillance report validation. All participating hospitals were familiar with the recommendations of the Clinical and Laboratory Standard Institute (CLSI) and European Committee on Antimicrobial Susceptibility Testing (EUCAST) on antimicrobial susceptibility testing (AST) data verification. Additionally, all participating hospitals were familiar with validating summary data by comparing the summary data automatically generated by the AMASS with manual calculations.

Users’ suggestions for improvement were received when the investigation team visited the participating hospitals. The users expressed a need for including other clinical specimen types (eg, urine samples) and a function to support data verification or provide a list of isolates with unusual AST profiles. This is because a data verification function is not currently available in many in-house LISs. Users also expressed the need for a way to import multiple data files and different data formats, particularly the long format (ie, each row represents an AST result obtained from each antibiotic against each isolate). This is because the data exported from automated machines for AST testing are commonly in the long format and it takes time to integrate the data from the automated AST machines and from disk diffusion AST results. Users also noted that the requirement to manually export data from the existing LIS can be tedious and time consuming to perform on a regular basis, and automation that can bypass the data extraction step would be useful.

## Discussion

We developed an open-access, offline, and easy-to-use application, AMASS, which allows hospitals, especially in LMICs, to automatically generate AMR surveillance reports from routinely collected electronic microbiology data. Seven hospitals used the AMASS, and shared their reports and summary data files by depositing them in an open-access data repository website. We propose that routine generation and sharing of AMR surveillance reports (ie, cumulative antimicrobial susceptibility reports or antibiograms) in open-access data repositories from hospitals with a microbiology laboratory should be supported worldwide [40].

The AMASS empowers data sharing by reducing the time and effort needed to prepare the summary data and reports without increasing the risks of breaching individual patient data confidentiality. This is because the AMASS can analyze the data and generate the reports promptly without needing to transfer raw data files to any party outside of the hospital. The autogenerated AMR surveillance reports are in PDF format, which can be reviewed and shared locally and immediately. This is important to improve the understanding of the local AMR burden and to inform local patient management. The PDF report and summary CSV files contain no individual patient-level information and can be shared with national and international organizations to support action plans on AMR. Any attempt to compare AMR surveillance reports among different hospitals should be done cautiously, as factors, including the type of hospital, blood culture utilization rates and practices, and patient characteristics, may influence the estimated prevalence and incidence rates of AMR infections [12-14]. An AMR surveillance report should clearly present the denominators used to calculate incidence rates. Selecting an appropriate denominator that represents the local setting is important for estimating the incidence rate of AMR, but remains challenging [41]. The AMASS calculates the total numbers of tested populations and uses them as denominators in section four and section five of the output report based on the WHO GLASS recommendations, and these denominators will not be available without data on negative cultures.

The AMASS uses currently recommended analytical approaches to generate the summary data and reports. The entire code is open access, verifiable, and modifiable. The AMASS can also be expanded and tailored based on local requirements. For instance, notifiable bacterial infections that are important for a local setting can be included by revising the R code provided in the AMASS package. Analytical methods can also be constantly updated and improved over time.

The AMASS is an add-on automatized report-generating tool that can be easily used, even by hospital users with limited information technology skills. It does not replace WHONET, LISs, quality assurance programs, or antimicrobial surveillance systems (including the WHO GLASS). The AMASS therefore differs from the “Macros and Excel Reports” function of WHONET, which allows users to regularly generate reports on screen and export data as Excel files using macro language (ie, a series of written study parameters generated automatically through the WHONET user interface grouped together) [19]. In contrast, the AMASS is designed to automatically compile summary results of multiple organisms into a single report. The current version of the AMASS does not support quality improvement or alert individuals to unexpected antibiogram results, which WHONET is capable of doing [19]. Thus, while WHONET is an appropriate choice for the main software package for microbiology laboratories in LMICs to capture and store microbiology data and to export data for the WHO GLASS [19], the AMASS provides important additional functionality [19].

Verifying individual AST results and validating summary data in the reports (including those generated by the AMASS) remain major barriers to the generation of accurate surveillance data on AMR. Only verified AST results on each patient’s isolate should be used in the data analysis [12-14]. WHONET and few LISs include functions that automatically check the results to ensure that they appear reasonable or alert users to confirm unusual results (eg, amikacin resistance coexisting with gentamicin and tobramycin susceptibility in *E. coli* is unusual, and data on such cases should be verified) [12-14,19]. In addition, it is important to validate the calculations of any analytical software used to generate the summary data and reports. We recommended that users of the AMASS perform manual validation (such as printing a line listing of all isolates of the species to cross-check with the reports), particularly when the program is used for the first time [12-14]. Users should also understand that the precision of the estimate is based on the sample size, which is illustrated by estimated confidence intervals. In addition, the representativeness of the summary cumulative AST data could be impacted by the sampling strategy used, particularly if blood culture is mainly performed for patients with treatment failure or prolonged hospitalization [11,12,14]. Nonetheless, generating AMR surveillance reports from existing microbiology databases could be the first step for hospitals in LMICs to understand and validate their own data, and support quality control programs at the local level.

Local hospitals must not be discredited because of the statistics in any AMR surveillance report, as possible criticism could be a barrier to data sharing [42]. Negative criticism of the data shared by local hospitals needs to be avoided. It is inevitable for errors to occur in any data set, especially in settings with limited resources and experience on quality assurance and data validation. Tools for data validation should be provided to local hospitals, and the statistics in an AMR report should act as a guide on the direction of improving data quality control, data validation, and infection prevention and control. Credit should be given to the hospitals that share their local AMR data and reports on an open-access data repository platform. Data repository platforms, such as figshare, allow flexibility on updating the data and revising the AMR surveillance reports with appropriate version controls by local hospitals. Publishing open-access AMR data on data repository platforms provides a digital object identifier (DOI) that should be used for data citation [43].

The AMASS has a number of limitations. First, the AMASS is not applicable for hospitals that only store data in paper forms. Second, the AMASS cannot work with raw microbiology data files that are not in a wide format or combine many files in multiple formats. For example, raw microbiology data file where each row contains data of each antibiotic susceptibility result for one single specimen. Third, the current version of the AMASS can only analyze microbiology data that include antimicrobial susceptibility test interpretive categories (susceptible, intermediate, and resistant) based on guidelines that the local hospital uses. Fourth, the AMASS cannot automatically validate the reliability of the data that are imported into the application and used for analysis. Data verification and quality checks will be included in future versions of the application. Fifth, the AMASS was tested with only few data sets that included non-English languages, and further testing on data with other languages may be needed. The current version of the AMASS has been tested on Windows 10 and 7 and may not work on other operating systems. Sixth, the current version of the AMASS cannot provide options to export the AMR surveillance report in different formats (ie, in document or text format). However, users can reuse the summary statistics, which are saved in CSV format under the ResultData folder. Finally, feedback from participating hospitals was not formally evaluated. Studies evaluating the barriers, facilitators, and utilities of supporting applications, such as the AMASS, to generate AMR surveillance reports are needed.

In conclusion, the AMASS can be used to support hospitals with microbiology laboratories in analyzing routinely collected data and generating reports with minimal resources and expertise required. This may empower individual hospitals to contribute to the understanding of and actions on AMR in local settings and maximize the utility of local data.

## Appendix

Multimedia Appendix 1 An overview of the flow of data processing and analyses performed by the AutoMated tool for Antimicrobial resistance Surveillance System (AMASS).

Multimedia Appendix 2 The list of pathogen-antibiotic combinations modified from the World Health Organization Global Antimicrobial Resistance Surveillance System priority list and included in the AutoMated tool for Antimicrobial resistance Surveillance System (AMASS).

Multimedia Appendix 3 The methods to estimate prevalence and incidence rates were based on the recommendation of the World Health Organization Global Antimicrobial Resistance Surveillance System.

Multimedia Appendix 4 Simulating hypothetical data sets.

Multimedia Appendix 5 How to run the AutoMated tool for Antimicrobial resistance Surveillance System (AMASS) using example data files.

Multimedia Appendix 6 How to configure the data dictionary files.

Multimedia Appendix 7 AMR surveillance report generated from the first example data set.

Multimedia Appendix 8 AMR surveillance report generated from the second example data set.

Multimedia Appendix 9 The list of participating hospitals.

Multimedia Appendix 10 How to export data from WHONET 5.6 (2019) in an Excel format that can be used by the AutoMated tool for Antimicrobial resistance Surveillance System (AMASS).

## Abbreviations

AMASS: AutoMated tool for Antimicrobial resistance Surveillance System
AMR: antimicrobial resistance
AST: antimicrobial susceptibility testing
CLSI: Clinical and Laboratory Standard Institute
EUCAST: European Committee on Antimicrobial Susceptibility Testing
GLASS: Global Antimicrobial Resistance Surveillance System
LIS: laboratory information system
LMIC: low and middle-income country
WHO: World Health Organization
