# Supplementary material for: Automating the Generation of Antimicrobial Resistance Surveillance Reports: Proof-of-Concept Study Involving Seven Hospitals in Seven Countries
Source: J Med Internet Res. 2020 Oct 2;22(10):e19762. doi: 10.2196/19762 (PMC7568216; doi:10.2196/19762)
Supplement: Multimedia Appendix 1 [file jmir_v22i10e19762_app1.docx]

**Multimedia Appendix 1.** An overview on the flow of data processing and analyses performed by AMASS.

**
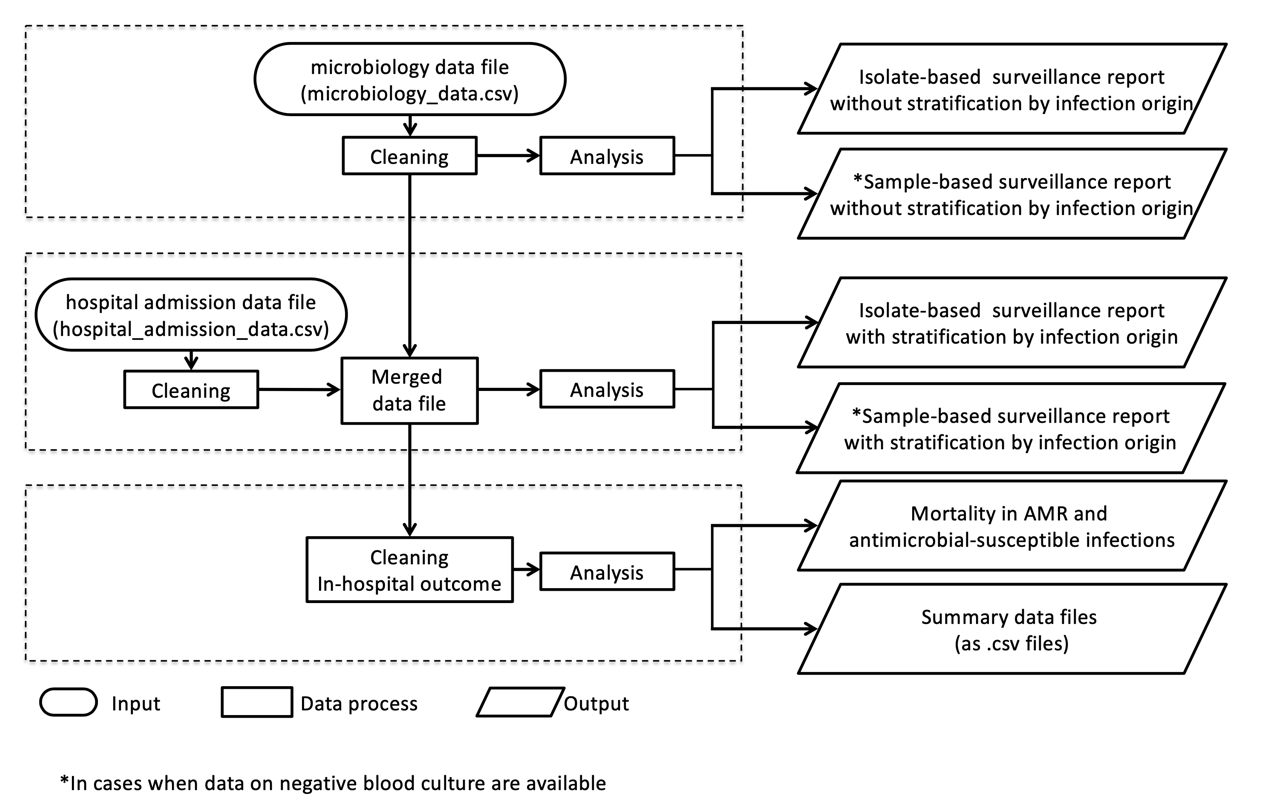
**
