# Supplementary material for: Automating the Generation of Antimicrobial Resistance Surveillance Reports: Proof-of-Concept Study Involving Seven Hospitals in Seven Countries
Source: J Med Internet Res. 2020 Oct 2;22(10):e19762. doi: 10.2196/19762 (PMC7568216; doi:10.2196/19762)
Supplement: Multimedia Appendix 2 [file jmir_v22i10e19762_app2.docx]

**Multimedia Appendix 2:** The list of pathogen-antibiotic combinations modified from the WHO GLASS priority list, and are included in the AutoMated tool for Antimicrobial resistance Surveillance System (AMASS).

| Type of Specimen | Pathogen | Antibacterial agents |
| --- | --- | --- |
| Blood | *Staphylococcus aureus* | Methicillin*  Vancomycin  Clindamycin |
| Blood | *Enterococcus* spp. | Ampicillin  Vancomycin  Teicoplanin  Linezolid  Daptomycin |
| Blood | *Streptococcus pneumoniae* | Penicillin G  Oxacillin  Co-trimoxazole  Ceftriaxone  Cefotaxime |
|  |  | Erythromycin  Clindamycin  Levofloxacin |
| Blood | *Salmonella* spp. | Ciprofloxacin  Levofloxacin  Ceftriaxone  Cefotaxime  Ceftazidime  Imipenem  Meropenem  Ertapenem  Doripenem |
|  |  |  |
| Blood | *Escherichia coli* | Gentamicin  Amikacin  Co-trimoxazole  Ampicillin  Ciprofloxacin  Levofloxacin  Cefpodoxime  Ceftriaxone  Cefotaxime  Ceftazidime  Cefepime  Imipenem  Meropenem  Ertapenem  Doripenem  Colistin |
| Blood | *Klebsiella pneumoniae* | Gentamicin  Amikacin  Co-trimoxazole  Ciprofloxacin  Levofloxacin  Cefpodoxime  Ceftriaxone  Cefotaxime  Ceftazidime  Cefepime  Imipenem  Meropenem  Ertapenem  Doripenem  Colistin |
| Blood | *Pseudomonas aeruginosa* | Ceftazidime  Ciprofloxacin  Piperacillin/tazobactam  Gentamicin  Amikacin  Imipenem  Meropenem  Doripenem  Colistin |
| Blood | *Acinetobacter baumannii* | Tigecycline  Minocycline  Gentamicin  Amikacin  Imipenem  Meropenem  Doripenem  Colistin |

*Oxacillin or cefoxitin may be used as a proxy if methicillin is not tested.
