# Supplementary material for: Automating the Generation of Antimicrobial Resistance Surveillance Reports: Proof-of-Concept Study Involving Seven Hospitals in Seven Countries
Source: J Med Internet Res. 2020 Oct 2;22(10):e19762. doi: 10.2196/19762 (PMC7568216; doi:10.2196/19762)
Supplement: Multimedia Appendix 3 [file jmir_v22i10e19762_app3.docx]

# Multimedia Appendix 3: The methods to estimate prevalence and incidence rates were based on the recommendation of the WHO GLASS.

**Proportion of AMR pathogen:**

| Proportion of patients with positive blood cultures who have resistant strains  (%, modified from WHO GLASS^11^) | = | Number of patients with blood culture positive for resistant strains of bacterial species under surveillance  (per pathogen per specimen per survey period) |
| --- | --- | --- |
|  |  | Total number of patients with blood culture positive for bacterial species under surveillance  (per pathogen per specimen per survey period) |
|  |  |  |

**Proportion of AMR pathogen stratified by origin of infection:**

| Proportion of patients with positive blood culture who have resistant strains per type of origin of infection  (%, modified from WHO GLASS^11^) | = | Number of patients with blood culture positive for resistant strains of bacterial species under surveillance  (per pathogen per specimen per survey period per origin of infection) |
| --- | --- | --- |
|  |  | Total number of patients with blood culture positive for bacterial species under surveillance  (per pathogen per specimen per survey period per origin of infection) |

**Incidence of AMR pathogen:**

| Frequency of patients with blood culture positive for pathogen  (per 100,000 tested patients, modified from WHO GLASS^11^) | = | Number of patients with blood culture positive for bacterial species in the population tested  during reporting period * 100,000  (per pathogen per specimen per survey period) |
| --- | --- | --- |
|  |  | Total number of patients with blood culture performed  during the reporting period |

| Frequency of patients with blood culture positive for resistant strains  (per 100,000 tested patients, modified from WHO GLASS^11^) | = | Number of patients with blood culture positive for resistant strains of bacterial species under surveillance  during reporting period * 100,000  (per pathogen per specimen per survey period) |
| --- | --- | --- |
|  |  | Total number of patients with blood culture performed  during the reporting period |

**Incidence of AMR pathogen stratified by origin of infection:**

| Frequency of patients with blood culture positive for pathogen  (per 100,000 tested patients, modified from WHO GLASS^11^) | = | Number of patients with blood culture positive for bacterial species in the population tested  during reporting period * 100,000  (per pathogen per specimen per survey period  per origin of infection) |
| --- | --- | --- |
|  |  | Total number of patients with the first blood culture performed **within the first two calendar days of admissions** during the reporting period |

| Frequency of patients with blood culture positive for resistant strains  (per 100,000 tested patients, modified from WHO GLASS^11^) | = | Number of patients with blood culture positive for resistant strains of bacterial species under surveillance  during reporting period * 100,000  (per pathogen per specimen per survey period  per origin of infection) |
| --- | --- | --- |
|  |  | Total number of patients with the first blood culture performed **after the first two calendar days of admission** during the reporting period |
