# Supplementary material for: Automating the Generation of Antimicrobial Resistance Surveillance Reports: Proof-of-Concept Study Involving Seven Hospitals in Seven Countries
Source: J Med Internet Res. 2020 Oct 2;22(10):e19762. doi: 10.2196/19762 (PMC7568216; doi:10.2196/19762)
Supplement: Multimedia Appendix 4 [file jmir_v22i10e19762_app4.docx]

# Multimedia Appendix 4: Simulating hypothetical data sets.

We generated a hypothetical data set for users to test AMASS and to visualize the data format required for the application. Two hypothetical data files (microbiology_data.xlsx and hospital_admission_data.xlsx) were generated using the summary statistics in the AMR surveillance report from Sunpasitthiprasong Hospital in 2015.^33^ The key variables generated were admission date, discharge date, gender, age (in year), in-hospital discharge outcome, specimen date, specimen type, organism isolated, and antibiogram results. The admission date was sampled from a uniform distribution ranged between 1^st^ January 2016 and 31^st^ December 2016. For bloodstream infections of community-origin, we assumed that the first blood specimen culture positive for the pathogen was collect on the first calendar day of hospitalization. For bloodstream infections of hospital-origin, we assumed that the first blood specimen culture positive for the pathogen was collected on the fourth day of hospitalization. The data was generated using STATA, version 15.1 (StataCorp LP, College station, Texas, USA).
