# Supplementary material for: Automating the Generation of Antimicrobial Resistance Surveillance Reports: Proof-of-Concept Study Involving Seven Hospitals in Seven Countries
Source: J Med Internet Res. 2020 Oct 2;22(10):e19762. doi: 10.2196/19762 (PMC7568216; doi:10.2196/19762)
Supplement: Multimedia Appendix 5 [file jmir_v22i10e19762_app5.docx]

**Multimedia Appendix 5**

<https://youtu.be/T_jvp7xiIs0>
