# Supplementary material for: Automating the Generation of Antimicrobial Resistance Surveillance Reports: Proof-of-Concept Study Involving Seven Hospitals in Seven Countries
Source: J Med Internet Res. 2020 Oct 2;22(10):e19762. doi: 10.2196/19762 (PMC7568216; doi:10.2196/19762)
Supplement: Multimedia Appendix 8 [file jmir_v22i10e19762_app8.pdf]

Antimicrobial Resistance (AMR) Surveillance Report

Hospital name: **Hypothetical Hospital**
Country name: **Hypothetical Country**

Data from:

01 Jan 2016 to 31 Dec 2016

Contact person: xxx_Can be changed in the dictionary_of_variable_data.csv_xxx
Contact address: xxx_Can be changed in the dictionary_of_variable_data.csv_xxx
Contact email: xxx@xxx.xxx
Generated on: 13 Apr 2020

Generated by

AutoMated tool for Antimicrobial resistance Surveillance System (AMASS)
Version 1.0 (released on February 1, 2019)

The AMASS application is available under the Creative Commons Attribution 4.0 International Public License (CC BY 4.0). The application can be downloaded at: http://www.amass.website

The AMASS application used microbiology_data and hospital_admission_data files that are stored in the same folder as the application (AMASS.bat) to generate this report.

The goal of the AMASS application is to enable hospitals with microbiology data available in electronic formats to analyze their own data and generate AMR surveillance reports promptly. If hospital admission date data are available, the reports will additionally be stratified by infection origin (community−origin or hospital−origin). If mortality data (such as patient discharge outcome data) are available, a report on mortality involving AMR infection will be added.

This automatically generated report has limitations, and requires users to understand those limitations and use the summary data in the report with careful interpretation.

A valid report could have local implications and much wider benefits if shared with national and international organizations.

This automatically generated report is under the jurisdiction of the hospital to copy, redistribute, and share with any individual or organization.

This automatically generated report contains no patient identifier, similar to standard reports on cumulative antimicrobial susceptibility.

For any query on AMASS, please contact:
Cherry Lim (cherry@tropmedres.ac) and
Direk Limmathurotsakul (direk@tropmedres.ac)

Suggested title for citation:
Antimicrobial resistance surveillance report, Hypothetical Hospital, Hypothetical Country, 01 Jan 2016 to 31 Dec 2016.

Content

Introduction 01

Section [1]: Data overview 03

Section [2]: Isolate−based surveillance report 05

Section [3]: Isolate−based surveillance report with stratification by infection origin 12

Section [4]: Sample−based surveillance report 24

Section [5]: Sample−based surveillance report with stratification by infection origin 27

Section [6]: Mortality involving AMR and antimicrobial−susuceptible infections 32

Methods 38

Acknowledgements 43

Introduction

Antimicrobial resistance (AMR) is a global health crisis [1]. The report by Lord Jim O'Neill estimated that 700,000 global deaths could be attributable to AMR in 2015, and projected that the annual death toll could reach 10 million by 2050 [1]. However, data of AMR surveillance from low and middle−income countries (LMICs) are scarce [1,2], and data of mortality associated with AMR infections are rarely available. A recent study estimated that 19,000 deaths are attributable to AMR infections in Thailand annually, using routinely available microbiological and hospital databases [3]. The study also proposed that hospitals in LMICs should utilize routinely available microbiological and hospital admission databases to generate reports on AMR surveillance systematically [3].

Reports on AMR surveillance can have a wide range of benefits [2]; including

- characterization of the frequency of resistance and organisms in different facilities and regions;
- prospective and retrospective information on emerging public health threats;
- evaluation and optimization of local and national standard treatment guidelines;
- evaluation of the impact of interventions beyond antimicrobial guidelines that aim to reduce AMR; and
- data sharing with national and international organizations to support decisions on resource allocation for interventions against AMR and to inform the implementation of action plans at national and global levels.

When reporting AMR surveillance results, it is generally recommended that (a) duplicate results of bacterial isolates are removed, and (b) reports are stratified by infection origin (community−origin or hospital−origin), if possible [2]. Many hospitals in LMICs lack time and resources needed to analyze the data (particularly to deduplicate data and to generate tables and figures), write the reports, and to release the data or reports [4].

AutoMated tool for Antimicrobial resistance Surveillance System (AMASS) was developed as an offline, open−access and easy−to−use application that allows a hospital to perform data analysis independently and generate isolate−based and sample−based surveillance reports stratified by infection origin from routinely collected electronic databases. The application was built in R, which is a free software environment. The application has been placed within a user−friendly interface that only requires the user to double−click on the application icon. The AMASS application can be downloaded at:
http://www.amass.website

Please note that the AMASS application and the automatically−generated report have limitations, and require readers to understand those limitations and review the reports and summary data carefully. We encourage the user of the AMASS application to perform manual validation (such as printing and listing isolates of the species to cross check with the reports), as recommended by Clinical and Laboratory Standards Insitute (CLSI) [5] and European Antimicrobial Resistance Surveillance Network (EUCAST) [6,7]. Moreover, it is important to note that the AMASS is an add−on automatized report generating tool and does not replace WHONET, Laboratory Information System (LIS), quality assurance programme, or antimicrobial surveillance systems (including the WHO GLASS).

References:

[1] O'Neill J. (2014) Antimicrobial resistance: tackling a crisis for the health and wealth of nations. Review on antimicrobial resistance. http://amr−review.org. (accessed on 3 Dec 2018).
[2] World Health Organization (2018) Global Antimicrobial Resistance Surveillance System (GLASS) Report. Early implantation 2016−2017. http://apps.who.int/iris/bitstream/handle/10665/259744/9789 241513449−eng.pdf. (accessed on 3 Dec 2018)
[3] Lim C., et al. (2016) Epidemiology and burden of multidrug−resistant bacterial infection in a developing country. Elife 5: e18082.
[4] Ashley EA, Shetty N, Patel J, et al. Harnessing alternative sources of antimicrobial resistance data to support surveillance in low−resource settings. J Antimicrob Chemother. 2019; 74(3):541−546.
[5] Clinical and Laboratory Standards Institute (CLSI). Analysis and Presentation of Cumulative Antimicrobial Susceptibility Test Data, 4th Edition. 2014. (accessed on 21 Jan 2020)
[6] European Antimicrobial Resistance Surveillance Network (EARS−Net). Antimicrobial resistance (AMR) reporting protocol 2018. (accessed on 21 Jan 2020)
[7] European Committee on Antimicrobial Susceptibility Testing (EUCAST). www.eucast.org (accessed on 21 Jan 2020)

Section [1]: Data overview

Introduction
An overview of the data detected by the AMASS application is generated by default. The summary is based on the raw data files saved within the same folder as the application file (AMASS.bat).

Please review and validate this section carefully before proceeds to the next section.

Results
The microbiology_data file (stored in the same folder as the application file) had:

56995 specimen data records with collection dates ranging from
01 Jan 2016 to ***31 Dec 2016***

The hospital_admission_data file (stored in the same folder as the application file) had:

109450 admission data records with hospital admission dates ranging from
01 Jan 2016 to ***31 Dec 2016***

Notes:
[1] If the periods of the data in microbiology_data and hospital_admission_data files are not similar, the automatically−generated report should be interpreted with caution. The AMASS generates the reports based on the available data.

Reporting period by months:

Data was stratified by month to assist detection of missing data, and verification of whether the month distribution of data records in microbiology_data file and hospital_ admission_data file reflected the microbiology culture frequency and admission rate of the hospital, respectively. For example if the number of specimens in the microbiology_data file reported below is lower than what is expected, please check the raw data file and data dictionary files.

Month	Number of specimen data records in microbiology_data file	Number of admission data records in hospital_admission_data file
January	*5114*	*8550*
February	*4698*	*8710*
March	*4697*	*8920*
April	*4423*	*8310*
May	*4684*	*8685*
June	*4625*	*8650*
July	*4531*	*8625*
August	*4990*	*9320*
September	*4662*	*8730*
October	*4953*	*9310*
November	*4894*	*9140*
December	*4724*	*8675*
Total:	*56995*	*109450*

Note:

[1] Additional general demographic data will be made available in the next version of the AMASS application.

Section [2]: Isolate−based surveillance report

Introduction

An isolate−based surveillance report is generated by default, even if the hospital_admission_data file is unavailable. This is to enable hospitals with only microbiology data available to utilize the de−duplication and report generation functions of AMASS.
This report is without stratification by origin of infection.

The report generated by the AMASS application version 1.0 includes only blood samples. The next version of AMASS will include other specimen types, including cerebrospinal fluid (CSF), urine, stool, and other specimens.

Organisms under this survey:

- *Staphylococcus aureus*
- *Enterococcus* spp.
- *Streptococcus pneumoniae*
- *Salmonella* spp.
- *Escherichia coli*
- *Klebsiella pneumoniae*
- *Pseudomonas aeruginosa*
- *Acinetobacter* spp.

Results

The microbiology_data file had:

Sample collection dates ranged from ***01 Jan 2016*** to ***31 Dec 2016***

Number of records of blood specimens collected within the above date range:

56995 blood specimens records

*Number of records of blood specimens with *negative culture (no growth):*

48992 blood specimens records

Number of records of blood specimens with culture positive for a microorganism:

8003 blood specimens records

Number of records of blood specimens with culture positive for organism under this survey:

3129 blood specimens records

The AMASS application de−duplicated the data by including only the first isolate per patient per specimen type per evaluation period as described in the method. The number of patients with positive samples is as follows:

**Organism**	**Number of records of blood specimens culture positive for the organism**	****Number of patients with blood culture positive for the organism (de−duplicated)**
Staphylococcus aureus	***417***	***212***
***Enterococcus* spp.**	***236***	***122***
Streptococcus pneumoniae	***75***	***38***
***Salmonella* spp.**	***123***	***62***
Escherichia coli	***1034***	***524***
Klebsiella pneumoniae	***543***	***279***
Pseudomonas aeruginosa	***224***	***116***
***Acinetobacter* spp.**	***477***	***244***
**Total:**	***3129***	***1597***

*The negative culture included data values specified as 'no growth' in the dictionary_for_ microbiology_data file (details on data dictionary files are in the method section) to represent specimens with negative culture for any microorganism.
**Only the first isolate for each patient per specimen type, per pathogen, and per evaluation period was included in the analysis.

The following figures and tables show the proportion of patients with blood culture positive for antimicrobial non−susceptible isolates.

Section [2]: Isolate−based surveillance report

Blood: *Staphylococcus aureus* (No. of patients = 212)

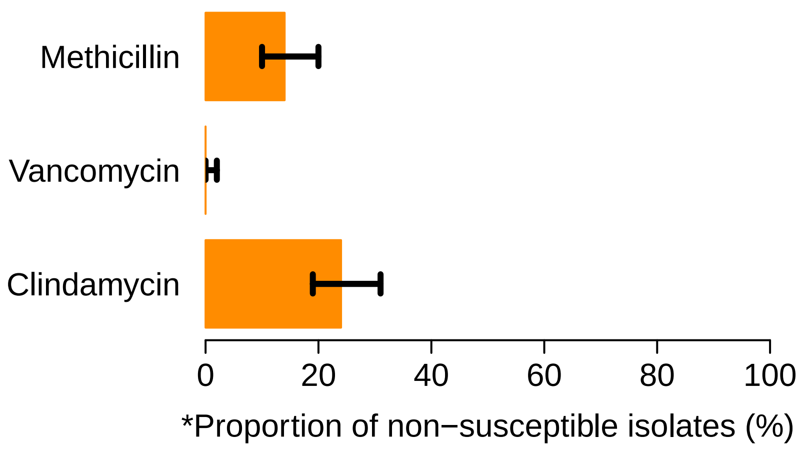

Antibiotic agent	% NS (n)	95% CI
Methicillin	14% (29/205)	10%−20%
Vancomycin	0% (0/205)	0%−2%
Clindamycin	24% (50/205)	19%−31%

Blood: *Enterococcus* spp. (No. of patients = 122)

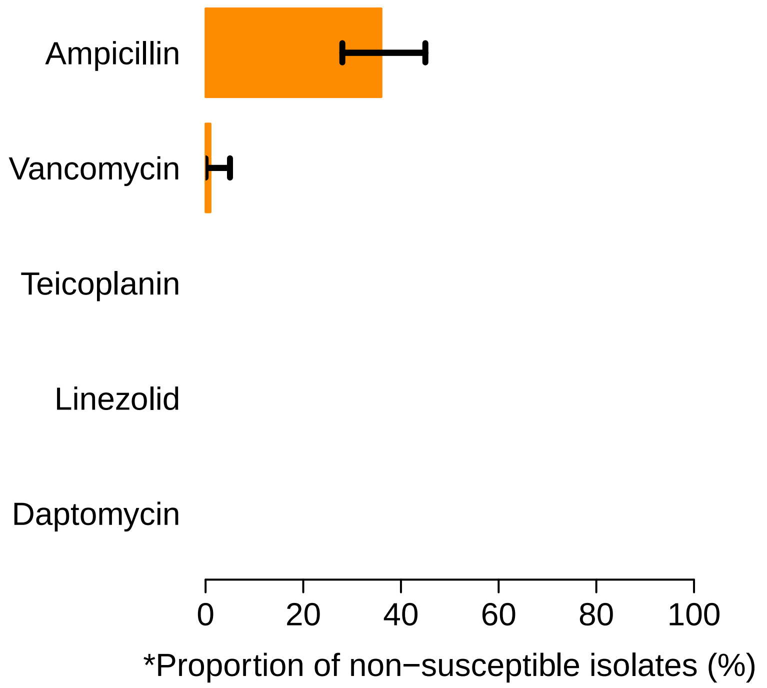

Antibiotic agent	% NS (n)	95% CI
Ampicillin	36% (41/114)	28%−45%
Vancomycin	1% (1/114)	0%−5%
Teicoplanin	NA	−
Linezolid	NA	−
Daptomycin	NA	−

*Proportion of non−susceptible isolates (% NS) represents the number of patients with blood culture positive for non−susceptible isolates (numerator) over the total number of patients with blood culture positive for the organism and the organism was tested for susceptibility against the antibiotic (denominator). The AMASS application de−duplicated the data by including only the first isolate per patient per specimen type per evaluation period. Grey bars indicate that testing with the antibiotic occurred for less than 70% of the total number of patients with blood culture positive for the organism.
CI = confidence interval; NA = Not available/reported/tested; Methicillin: methicillin, oxacillin, or cefoxitin

Section [2]: Isolate−based surveillance report

Blood: *Streptococcus pneumoniae*

(No. of patients = 38)

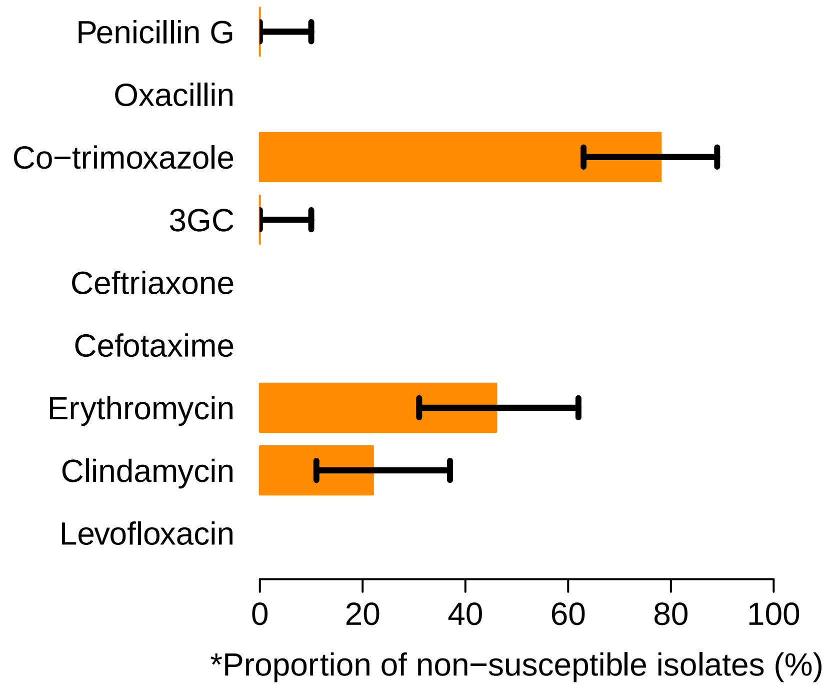

Antibiotic agent	% NS (n)	95% CI
Penicillin G	0% (0/36)	0%−10%
Oxacillin	NA	−
Co−trimoxazole	78% (29/37)	63%−89%
3GC	0% (0/36)	0%−10%
Ceftriaxone	NA	−
Cefotaxime	NA	−
Erythromycin	46% (17/37)	31%−62%
Clindamycin	22% (8/37)	11%−37%
Levofloxacin	NA	−

Blood: *Salmonella* spp.

(No. of patients = 62)

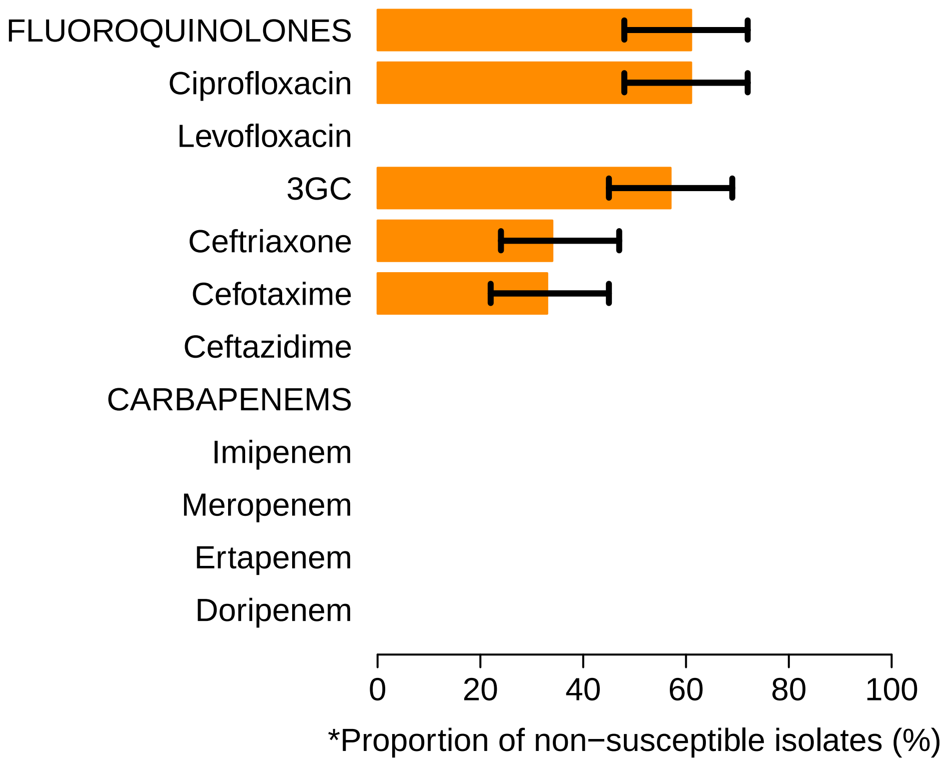

Antibiotic agent	% NS (n)	95% CI
FLUOROQUINOLONES	61% (37/61)	48%−72%
Ciprofloxacin	61% (37/61)	48%−72%
Levofloxacin	NA	−
3GC	57% (35/61)	45%−69%
Ceftriaxone	34% (21/61)	24%−47%
Cefotaxime	33% (20/61)	22%−45%
Ceftazidime	NA	−
CARBAPENEMS	NA	−
Imipenem	NA	−
Meropenem	NA	−
Ertapenem	NA	−
Doripenem	NA	−

*Proportion of non−susceptible isolates (% NS) represents the number of patients with blood culture positive for non−susceptible isolates (numerator) over the total number of patients with blood culture positive for the organism and the organism was tested for susceptibility against the antibiotic (denominator). The AMASS application de−duplicated the data by including only the first isolate per patient per specimen type per evaluation period. Grey bars indicate that testing with the antibiotic occurred for less than 70% of the total number of blood culture positive for the organism.
CI = confidence interval; NA = Not available/reported/tested; 3GC = 3rd−generation cephalosporin;
FLUOROQUINOLONES: ciprofloxacin or levofloxacin; CARBAPENEMS: imipenem, meropenem, ertapenem or doripenem

Section [2]: Isolate−based surveillance report

Blood: ***Escherichia coli*** ***(No. of patients = 524)***

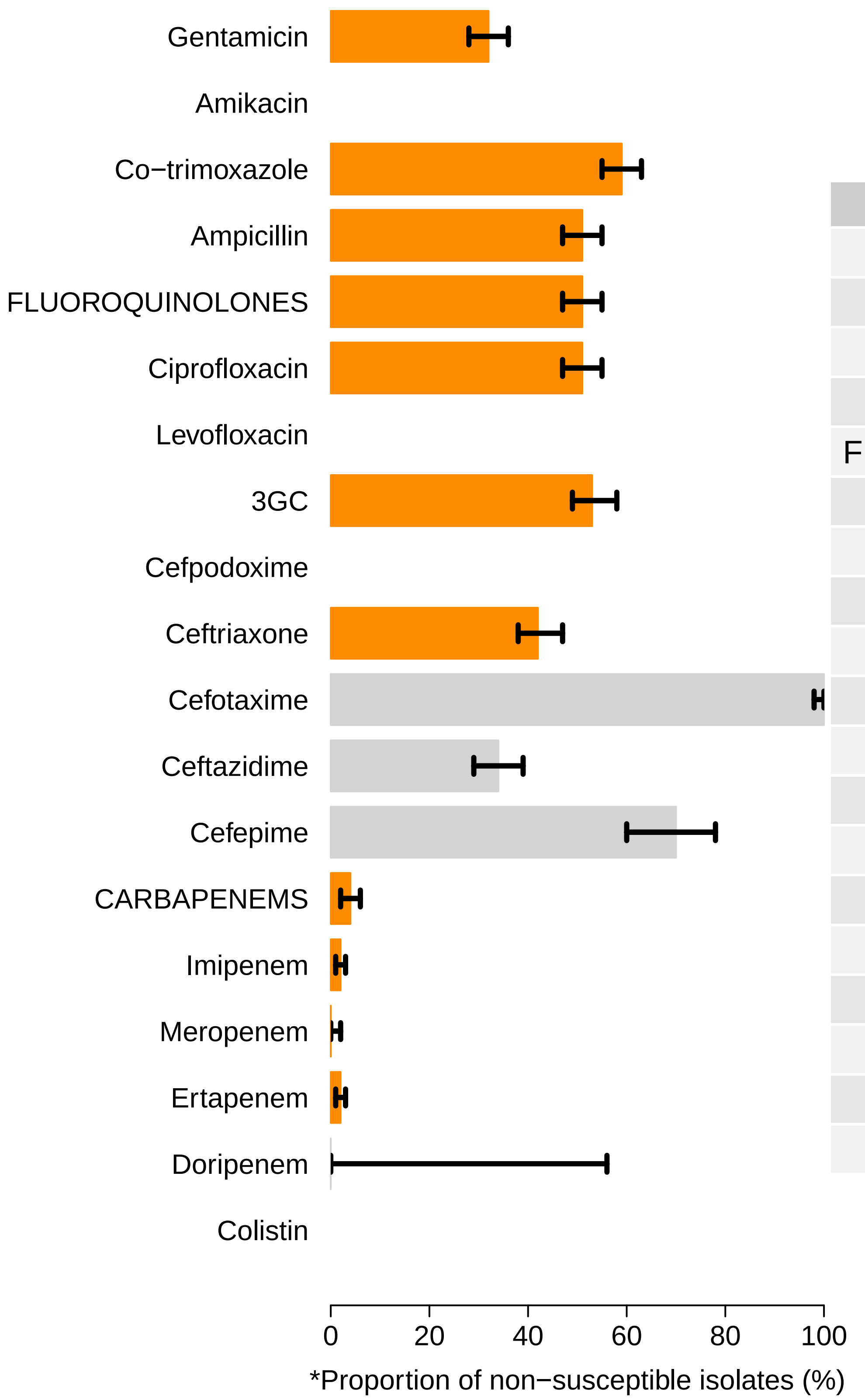

Antibiotic agent	% NS (n)	95% CI
Gentamicin	32% (163/510)	28%−36%
Amikacin	NA	−
Co−trimoxazole	59% (300/510)	55%−63%
Ampicillin	51% (261/510)	47%−55%
FLUOROQUINOLONES	51% (261/510)	47%−55%
Ciprofloxacin	51% (261/510)	47%−55%
Levofloxacin	NA	−
3GC	53% (272/510)	49%−58%
Cefpodoxime	NA	−
Ceftriaxone	42% (216/510)	38%−47%
Cefotaxime	100% (216/216)	98%−100%
Ceftazidime	34% (97/287)	29%−39%
Cefepime	70% (67/96)	60%−78%
CARBAPENEMS	4% (19/510)	2%−6%
Imipenem	2% (9/510)	1%−3%
Meropenem	0% (2/408)	0%−2%
Ertapenem	2% (8/510)	1%−3%
Doripenem	0% (0/3)	0%−56%
Colistin	NA	−

*Proportion of non−susceptible isolates (% NS) represents the number of patients with blood culture positive for non−susceptible isolates (numerator) over the total number of patients with blood culture positive for the organism and the organism was tested for susceptibility against the antibiotic (denominator). The AMASS application de−duplicated the data by including only the first isolate per patient per specimen type per evaluation period. Grey bars indicate that testing with the antibiotic occurred for less than 70% of the total number of blood culture positive for the organism.
CI = confidence interval; NA = Not available/reported/tested; 3GC = 3rd−generation cephalosporin; FLUOROQUINOLONES: ciprofloxacin or levofloxacin; CARBAPENEMS: imipenem, meropenem, ertapenem or doripenem

Section [2]: Isolate−based surveillance report

Blood: ***Klebsiella pneumoniae*** ***(No. of patients = 279)***

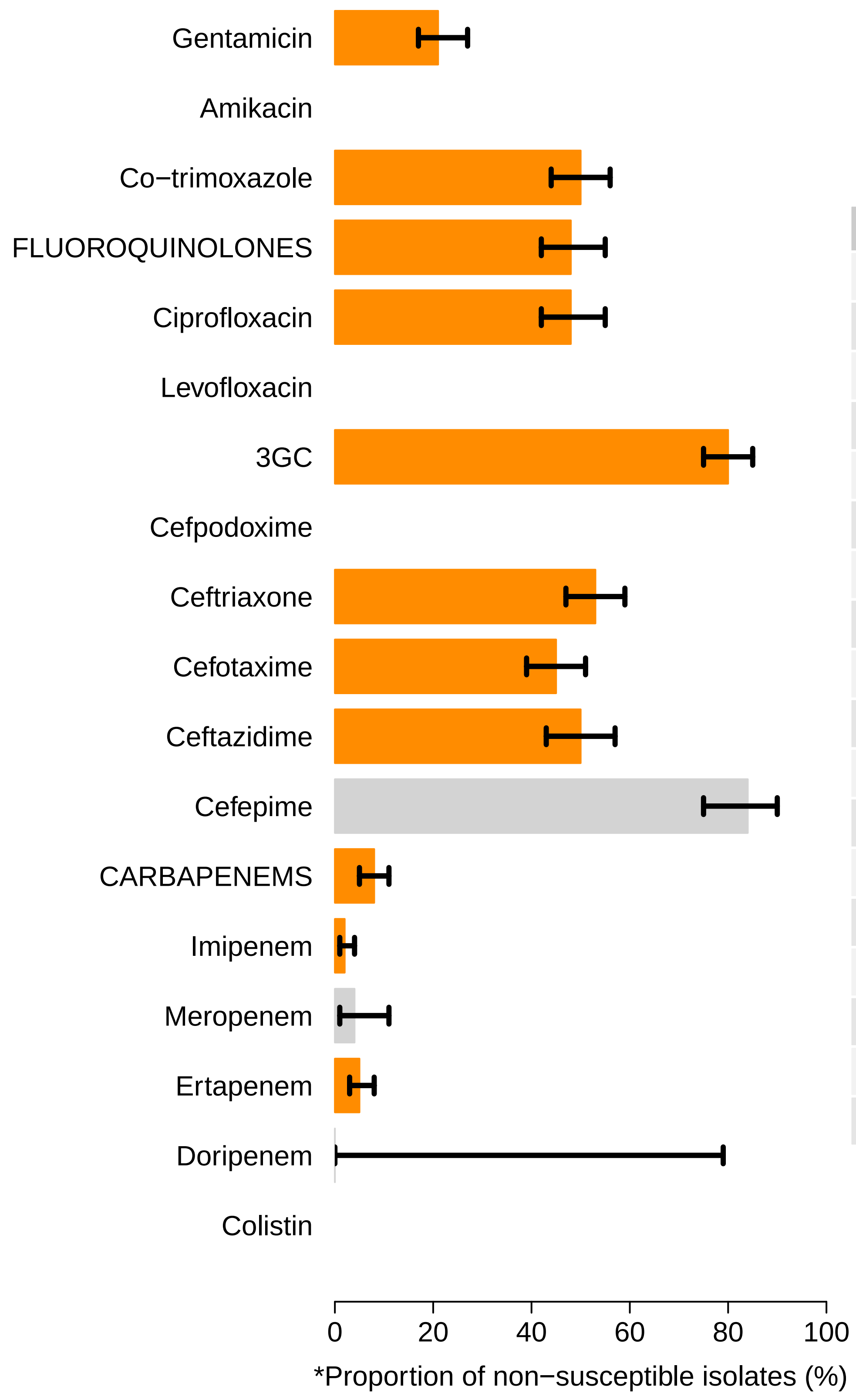

Antibiotic agent	% NS (n)	95% CI
Gentamicin	21% (56/264)	17%−27%
Amikacin	NA	−
Co−trimoxazole	50% (133/264)	44%−56%
FLUOROQUINOLONES	48% (104/215)	42%−55%
Ciprofloxacin	48% (104/215)	42%−55%
Levofloxacin	NA	−
3GC	80% (212/264)	75%−85%
Cefpodoxime	NA	−
Ceftriaxone	53% (139/264)	47%−59%
Cefotaxime	45% (119/264)	39%−51%
Ceftazidime	50% (102/204)	43%−57%
Cefepime	84% (72/86)	75%−90%
CARBAPENEMS	8% (20/264)	5%−11%
Imipenem	2% (4/264)	1%−4%
Meropenem	4% (3/73)	1%−11%
Ertapenem	5% (13/264)	3%−8%
Doripenem	0% (0/1)	0%−79%
Colistin	NA	−

*Proportion of non−susceptible isolates (% NS) represents the number of patients with blood culture positive for non−susceptible isolates (numerator) over the total number of patients with blood culture positive for the organism and the organism was tested for susceptibility against the antibiotic (denominator). The AMASS application de−duplicated the data by including only the first isolate per patient per specimen type per evaluation period. Grey bars indicate that testing with the antibiotic occurred for less than 70% of the total number of blood culture positive for the organism.
CI = confidence interval; NA = Not available/reported/tested; 3GC = 3rd−generation cephalosporin; FLUOROQUINOLONES: ciprofloxacin or levofloxacin; CARBAPENEMS: imipenem, meropenem, ertapenem or doripenem

Section [2]: Isolate−based surveillance report

Blood: ***Pseudomonas aeruginosa***

(No. of patients = 116)

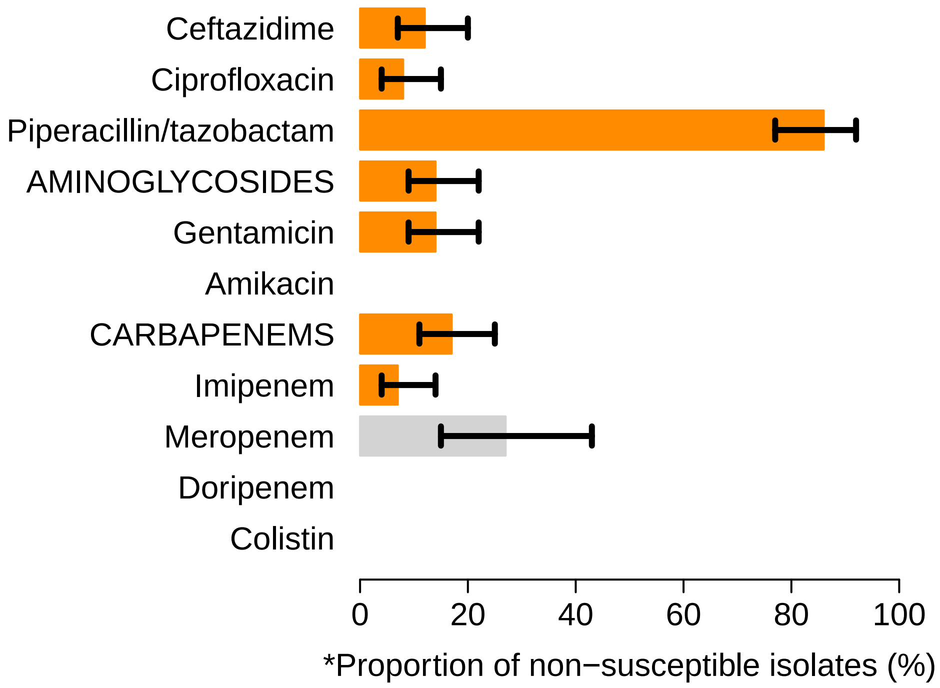

Antibiotic agent	% NS (n)	95% CI
Ceftazidime	12% (13/108)	7%−20%
Ciprofloxacin	8% (9/108)	4%−15%
Piperacillin/tazobactam	86% (74/86)	77%−92%
AMINOGLYCOSIDES	14% (15/108)	9%−22%
Gentamicin	14% (15/108)	9%−22%
Amikacin	NA	−
CARBAPENEMS	17% (18/108)	11%−25%
Imipenem	7% (8/108)	4%−14%
Meropenem	27% (10/37)	15%−43%
Doripenem	NA	−
Colistin	NA	−

Blood: ***Acinetobacter* spp.**

(No. of patients = 244)

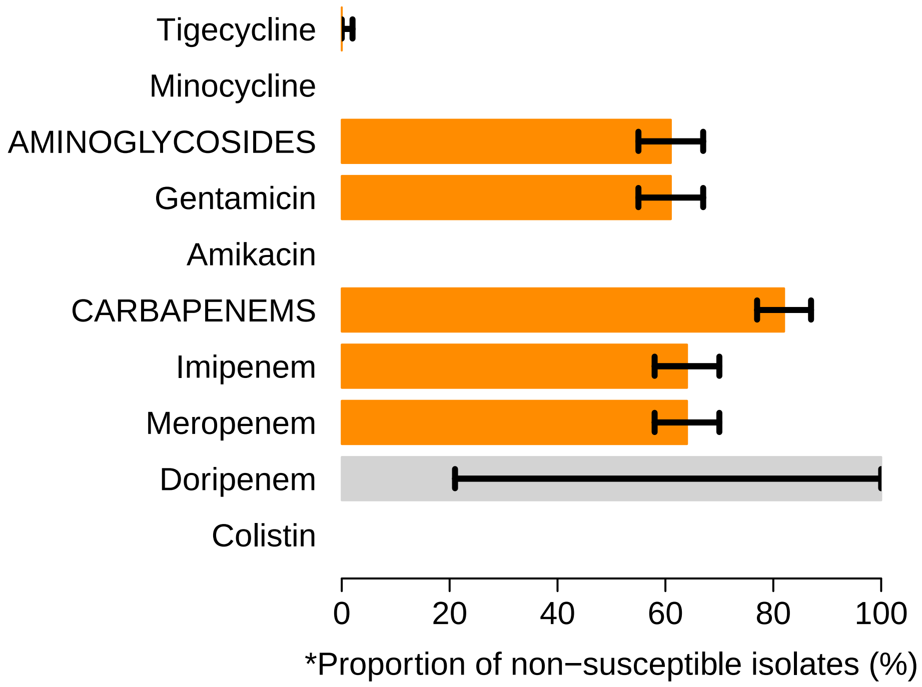

Antibiotic agent	% NS (n)	95% CI
Tigecycline	0% (0/233)	0%−2%
Minocycline	NA	−
AMINOGLYCOSIDES	61% (143/233)	55%−67%
Gentamicin	61% (143/233)	55%−67%
Amikacin	NA	−
CARBAPENEMS	82% (192/233)	77%−87%
Imipenem	64% (149/233)	58%−70%
Meropenem	64% (150/233)	58%−70%
Doripenem	100% (1/1)	21%−100%
Colistin	NA	−

*Proportion of non−susceptible isolates (% NS) represents the number of patients with blood culture positive for non−susceptible isolates (numerator) over the total number of patients with blood culture positive for the organism and the organism was tested for susceptibility against the antibiotic (denominator). The AMASS application de−duplicated the data by including only the first isolate per patient per specimen type per evaluation period. Grey bars indicate that testing with the antibiotic occurred for less than 70% of the total number of blood culture positive for the organism.
CI = confidence interval; NA = Not available/reported/tested; AMINOGLYCOSIDES: either gentamicin or amikacin; CARBAPENEMS: imipenem, meropenem, ertapenem or doripenem

Section [3]: Isolate−based surveillance report with stratification by infection origin

Introduction

An isolate−based surveillance report with stratification by origin of infection is generated only if admission date data are available in the raw data file(s) with the appropriate specification in the data dictionaries.

Stratification by origin of infection is used as a proxy to define where the bloodstream infection (BSI) was contracted (hospital versus community).

The definitions of infection origin proposed by the WHO GLASS are used. In brief, community−origin BSI is defined as patients in the hospital for less than or equal to two calendar days when the first specimen culture postive for the pathogen was taken. Hospital−origin BSI is defined as patients admitted for more than two calendar days when the first specimen culture positive for the pathogen was taken.

Results:

The data included in the analysis to generate the report had:

Sample collection dates ranged from ***01 Jan 2016*** to ***31 Dec 2016***

**Number of patients with blood culture positive for pathogen under the survey:*

1597 patients

***Number of patients with community−origin BSI:*

929 patients

***Number of patients with hospital−origin BSI:*

603 patients

****Number of patients with unknown infection of origin status:*

65 patients

Organism	Number of patients with blood culture positive for the organism	Community −origin**	Hospital −origin**	Unknown origin***
Staphylococcus aureus	212	148	57	7
Enterococcus spp.	122	59	55	8
Streptococcus pneumoniae	38	37	0	1
Salmonella spp.	62	52	9	1
Escherichia coli	524	397	113	14
Klebsiella pneumoniae	279	140	124	15
Pseudomonas aeruginosa	116	35	73	8
Acinetobacter spp.	244	61	172	11
Total:	1597	929	603	65

Note:
NA= Not applicable (hospital admission date or infection origin data are not available)

*Only the first isolate for each patient per specimen type per pathogen under the reporting period is included in the analysis. Please refer to Section [2] for details on how this number was calculated from the raw microbiology_data file.
**The definitions of infection origin proposed by the WHO GLASS is used. In brief, community−origin BSI was defined as patients in the hospital for less than or equal to two calendar days when the first blood culture positive for the pathogen was taken. Hospital−origin BSI was defined as patients admitted for more than two calendar days when the first specimen culture positive for the pathogen was taken.
Please refer to the 'Methods' section for more details on the definitions used.
***Unknown origin could be because admission date data are not available or the patient was not hospitalised.

The following figures and tables below show the proportion of patients with blood culture positive for antimicrobial non−susceptible isolates stratified by infection of origin.

Section [3]: Isolate−based surveillance report with stratification by infection origin

Blood: ***Staphylococcus aureus*** **Community−origin** ***(No. of patients = 148)***

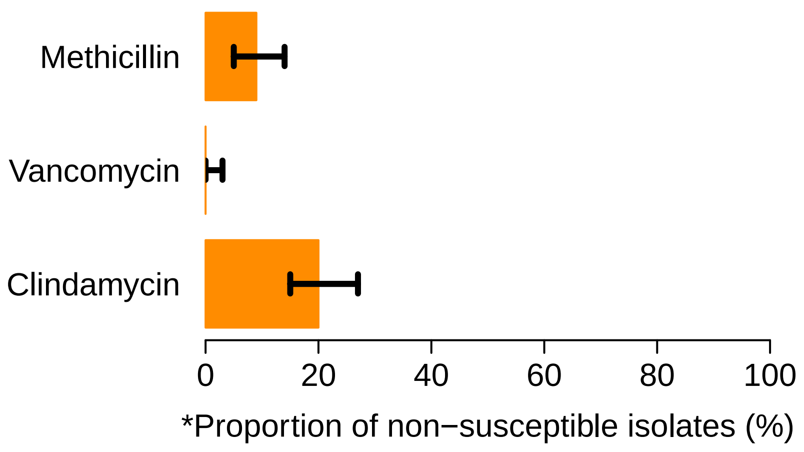

Antibiotic agent	% NS (n)	95% CI
Methicillin	9% (13/148)	5%−14%
Vancomycin	0% (0/148)	0%−3%
Clindamycin	20% (30/148)	15%−27%

Blood: ***Staphylococcus aureus*** **Hospital−origin** ***(No. of patients = 57)***

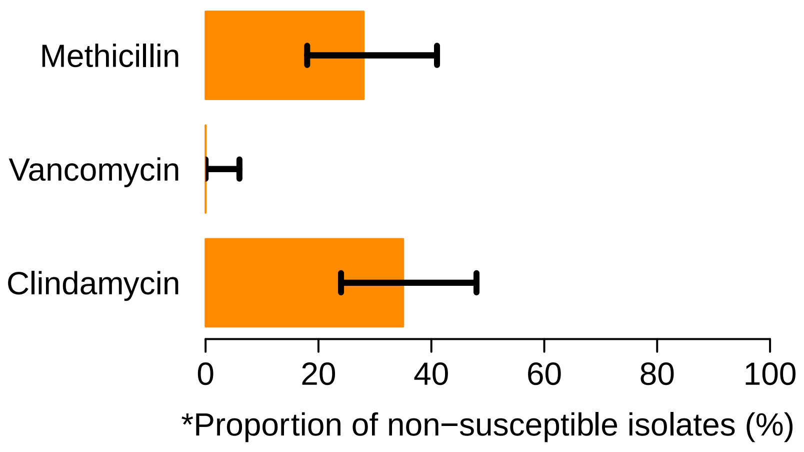

Antibiotic agent	% NS (n)	95% CI
Methicillin	28% (16/57)	18%−41%
Vancomycin	0% (0/57)	0%−6%
Clindamycin	35% (20/57)	24%−48%

*Proportion of non−susceptible isolates (% NS) represents the number of patients with blood culture positive for non−susceptible isolates (numerator) over the total number of patients with blood culture positive for the organism and the organism was tested for susceptibility against the antibiotic (denominator). The AMASS application de−duplicated the data by including only the first isolate per patient per specimen type per evaluation period. Grey bars indicate that testing with the antibiotic occurred for less than 70% of the total number of patients with blood culture positive for the organism.
CI = confidence interval; NA = Not available/reported/tested; Methicillin: methicillin, oxacillin, or cefoxitin

Section [3]: Isolate−based surveillance report with stratification by infection origin

Blood: *Enterococcus* spp. Community−origin (No. of patients = 59)

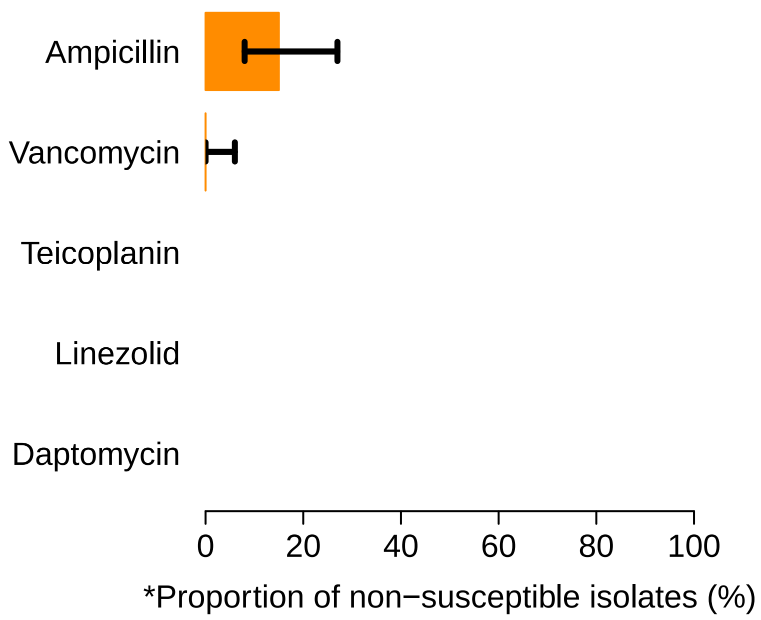

Antibiotic agent	% NS (n)	95% CI
Ampicillin	15% (9/59)	8%−27%
Vancomycin	0% (0/59)	0%−6%
Teicoplanin	NA	−
Linezolid	NA	−
Daptomycin	NA	−

Blood: *Enterococcus* spp. Hospital−origin (No. of patients = 55)

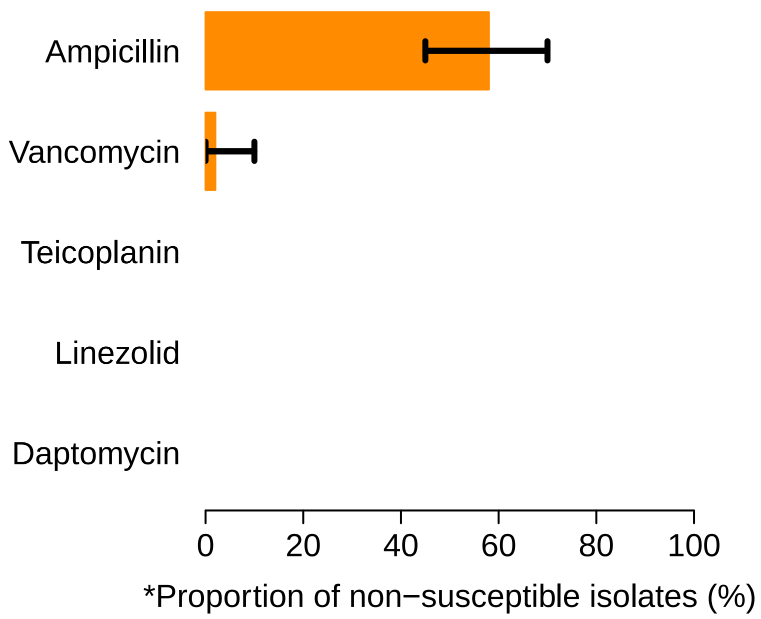

Antibiotic agent	% NS (n)	95% CI
Ampicillin	58% (32/55)	45%−70%
Vancomycin	2% (1/55)	0%−10%
Teicoplanin	NA	−
Linezolid	NA	−
Daptomycin	NA	−

*Proportion of non−susceptible isolates (% NS) represents the number of patients with blood culture positive for non−susceptible isolates (numerator) over the total number of patients with blood culture positive for the organism and the organism was tested for susceptibility against the antibiotic (denominator). The AMASS application de−duplicated the data by including only the first isolate per patient per specimen type per evaluation period. Grey bars indicate that testing with the antibiotic occurred for less than 70% of the total number of patients with blood culture positive for the organism.
CI = confidence interval; NA = Not available/reported/tested; Methicillin: methicillin, oxacillin, or cefoxitin

Section [3]: Isolate−based surveillance report with stratification by infection origin

Blood: *Streptococcus pneumoniae* Community−origin ***(No. of patients = 37)***

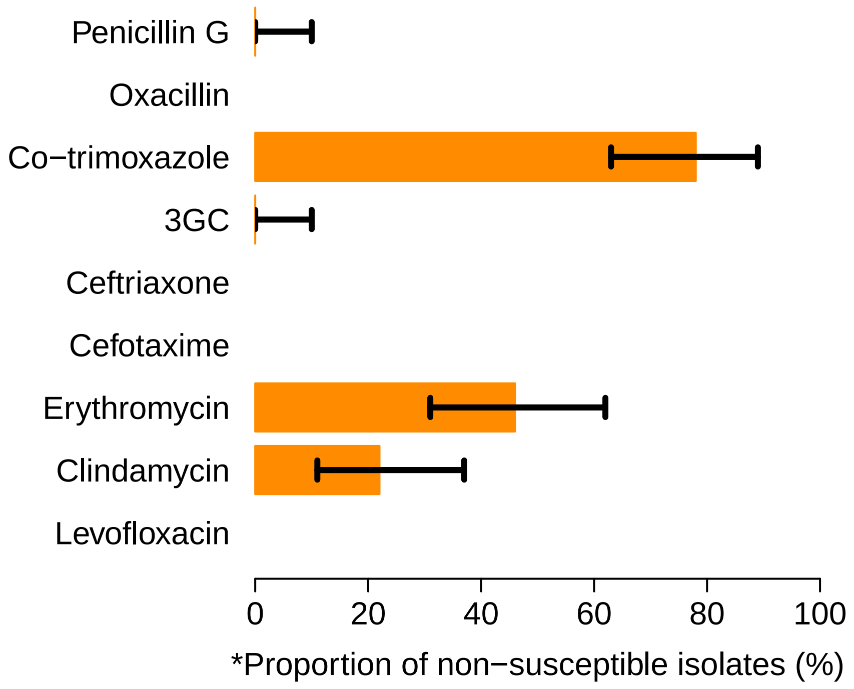

Antibiotic agent	% NS (n)	95% CI
Penicillin G	0% (0/36)	0%−10%
Oxacillin	NA	−
Co−trimoxazole	78% (29/37)	63%−89%
3GC	0% (0/36)	0%−10%
Ceftriaxone	NA	−
Cefotaxime	NA	−
Erythromycin	46% (17/37)	31%−62%
Clindamycin	22% (8/37)	11%−37%
Levofloxacin	NA	−

Blood: *Streptococcus pneumoniae* Hospital−origin ***(No. of patients = 0)***

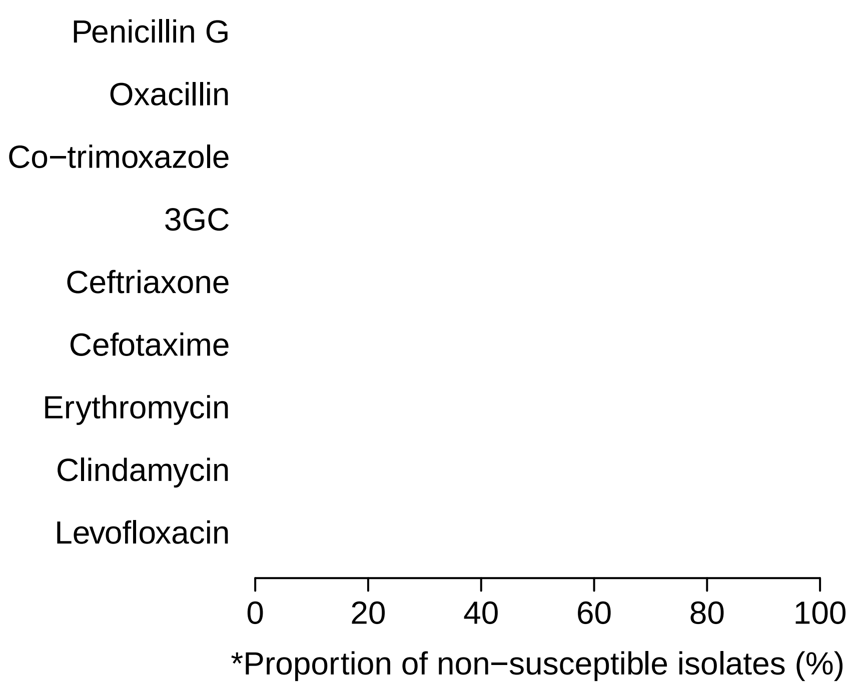

Antibiotic agent	% NS (n)	95% CI
Penicillin G	NA	−
Oxacillin	NA	−
Co−trimoxazole	NA	−
3GC	NA	−
Ceftriaxone	NA	−
Cefotaxime	NA	−
Erythromycin	NA	−
Clindamycin	NA	−
Levofloxacin	NA	−

*Proportion of non−susceptible isolates (% NS) represents the number of patients with blood culture positive for non−susceptible isolates (numerator) over the total number of patients with blood culture positive for the organism and the organism was tested for susceptibility against the antibiotic (denominator). The AMASS application de−duplicated the data by including only the first isolate per patient per specimen type per evaluation period. Grey bars indicate that testing with the antibiotic occurred for less than 70% of the total number of blood culture positive for the organism.
CI = confidence interval; NA = Not available/reported/tested; 3GC = 3rd−generation cephalosporin; FLUOROQUINOLONES: ciprofloxacin or levofloxacin; CARBAPENEMS: imipenem, meropenem, ertapenem or doripenem

Section [3]: Isolate−based surveillance report with stratification by infection origin

Blood: *Salmonella* spp. Community−origin ***(No. of patients = 52)***

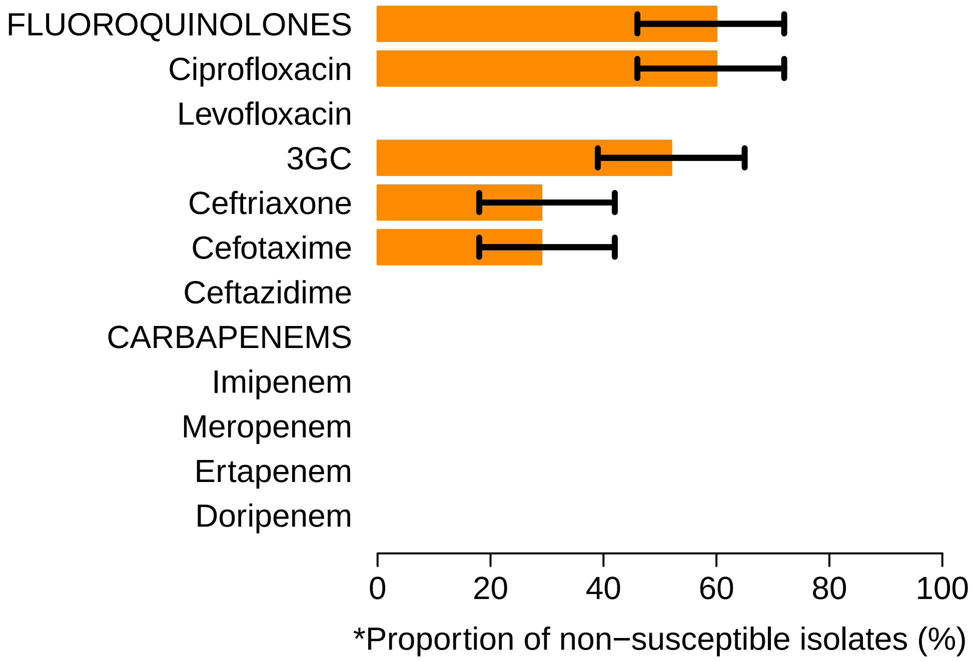

Antibiotic agent	% NS (n)	95% CI
FLUOROQUINOLONES	60% (31/52)	46%−72%
Ciprofloxacin	60% (31/52)	46%−72%
Levofloxacin	NA	−
3GC	52% (27/52)	39%−65%
Ceftriaxone	29% (15/52)	18%−42%
Cefotaxime	29% (15/52)	18%−42%
Ceftazidime	NA	−
CARBAPENEMS	NA	−
Imipenem	NA	−
Meropenem	NA	−
Ertapenem	NA	−
Doripenem	NA	−

Blood: *Salmonella* spp. Hospital−origin ***(No. of patients = 9)***

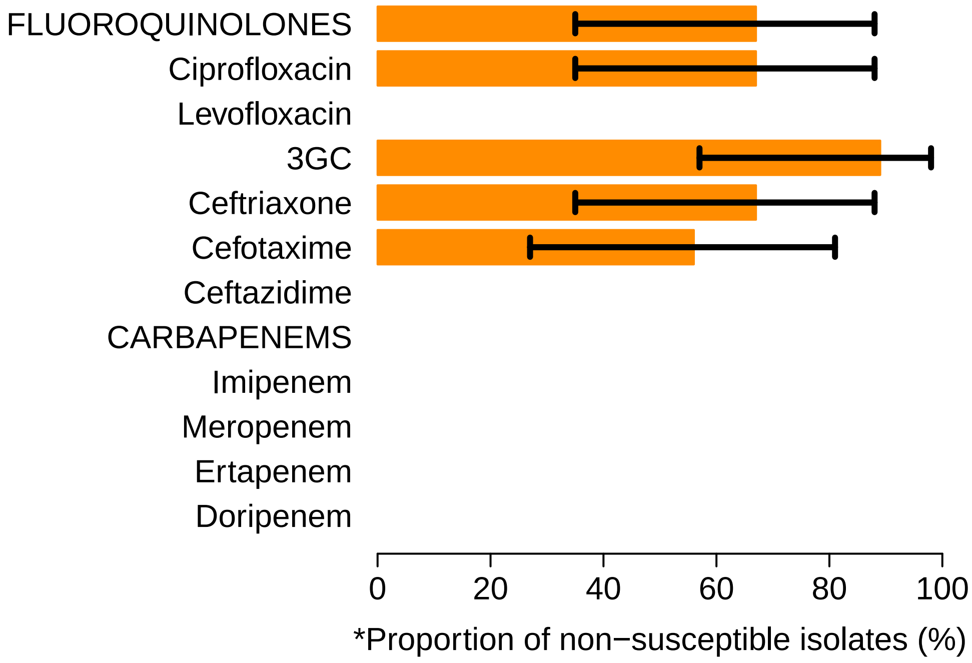

Antibiotic agent	% NS (n)	95% CI
FLUOROQUINOLONES	67% (6/9)	35%−88%
Ciprofloxacin	67% (6/9)	35%−88%
Levofloxacin	NA	−
3GC	89% (8/9)	57%−98%
Ceftriaxone	67% (6/9)	35%−88%
Cefotaxime	56% (5/9)	27%−81%
Ceftazidime	NA	−
CARBAPENEMS	NA	−
Imipenem	NA	−
Meropenem	NA	−
Ertapenem	NA	−
Doripenem	NA	−

*Proportion of non−susceptible isolates (% NS) represents the number of patients with blood culture positive for non−susceptible isolates (numerator) over the total number of patients with blood culture positive for the organism and the organism was tested for susceptibility against the antibiotic (denominator). The AMASS application de−duplicated the data by including only the first isolate per patient per specimen type per evaluation period. Grey bars indicate that testing with the antibiotic occurred for less than 70% of the total number of blood culture positive for the organism.
CI = confidence interval; NA = Not available/reported/tested; 3GC = 3rd−generation cephalosporin; FLUOROQUINOLONES: ciprofloxacin or levofloxacin; CARBAPENEMS: imipenem, meropenem, ertapenem or doripenem

Section [3]: Isolate−based surveillance report with stratification by infection origin

Blood: *Escherichia coli*

Community−origin ***(No. of patients = 397)***

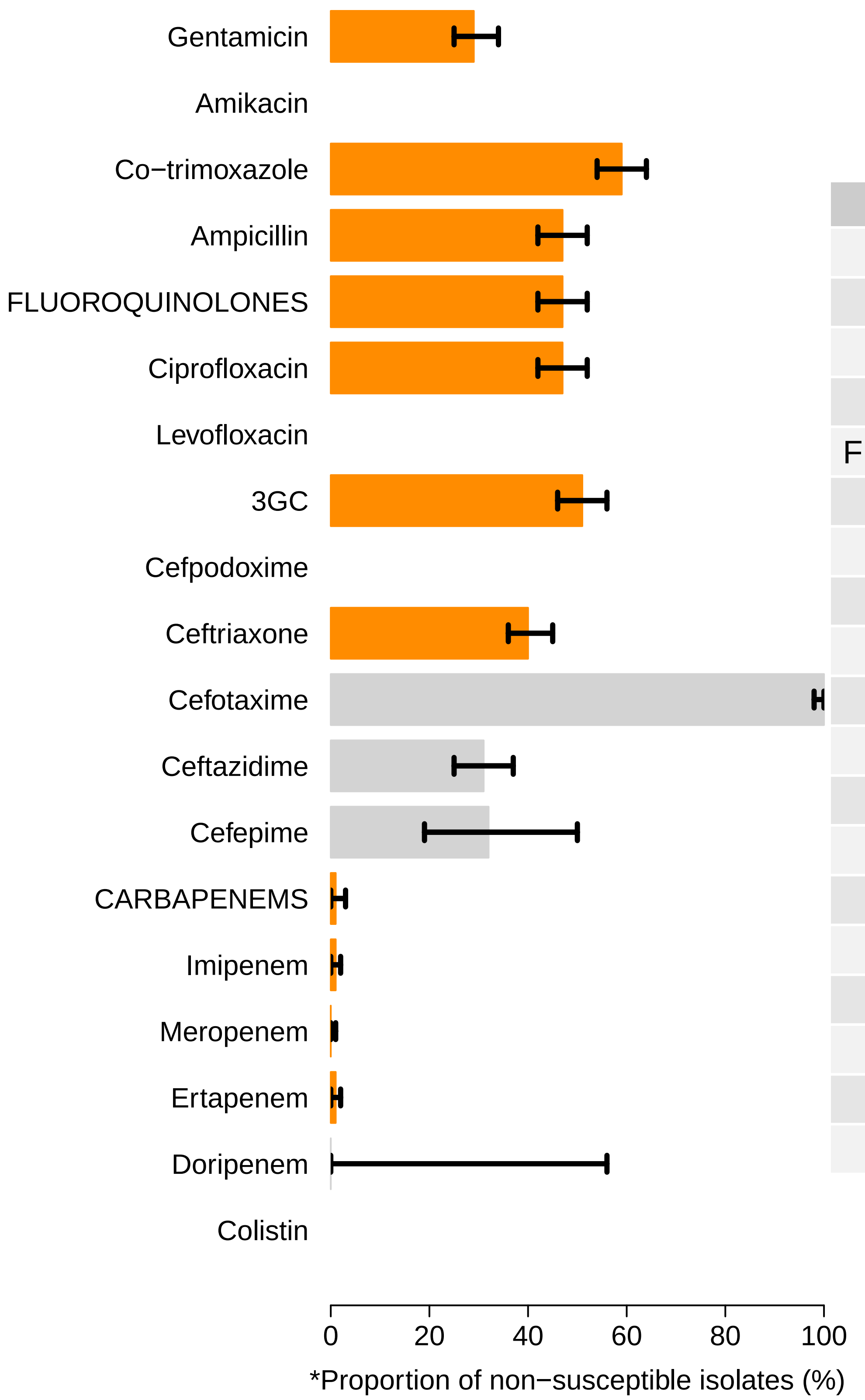

Antibiotic agent	% NS (n)	95% CI
Gentamicin	29% (116/397)	25%−34%
Amikacin	NA	−
Co−trimoxazole	59% (235/397)	54%−64%
Ampicillin	47% (185/397)	42%−52%
FLUOROQUINOLONES	47% (185/397)	42%−52%
Ciprofloxacin	47% (185/397)	42%−52%
Levofloxacin	NA	−
3GC	51% (203/397)	46%−56%
Cefpodoxime	NA	−
Ceftriaxone	40% (160/397)	36%−45%
Cefotaxime	100% (160/160)	98%−100%
Ceftazidime	31% (65/211)	25%−37%
Cefepime	32% (10/31)	19%−50%
CARBAPENEMS	1% (4/397)	0%−3%
Imipenem	1% (2/397)	0%−2%
Meropenem	0% (0/319)	0%−1%
Ertapenem	1% (2/397)	0%−2%
Doripenem	0% (0/3)	0%−56%
Colistin	NA	−

*Proportion of non−susceptible isolates (% NS) represents the number of patients with blood culture positive for non−susceptible isolates (numerator) over the total number of patients with blood culture positive for the organism and the organism was tested for susceptibility against the antibiotic (denominator). The AMASS application de−duplicated the data by including only the first isolate per patient per specimen type per evaluation period. Grey bars indicate that testing with the antibiotic occurred for less than 70% of the total number of blood culture positive for the organism.
CI = confidence interval; NA = Not available/reported/tested; 3GC = 3rd−generation cephalosporin; FLUOROQUINOLONES: ciprofloxacin or levofloxacin; CARBAPENEMS: imipenem, meropenem, ertapenem or doripenem

Section [3]: Isolate−based surveillance report with stratification by infection origin

Blood: ***Escherichia coli*** **Hospital−origin** ***(No. of patients = 113)***

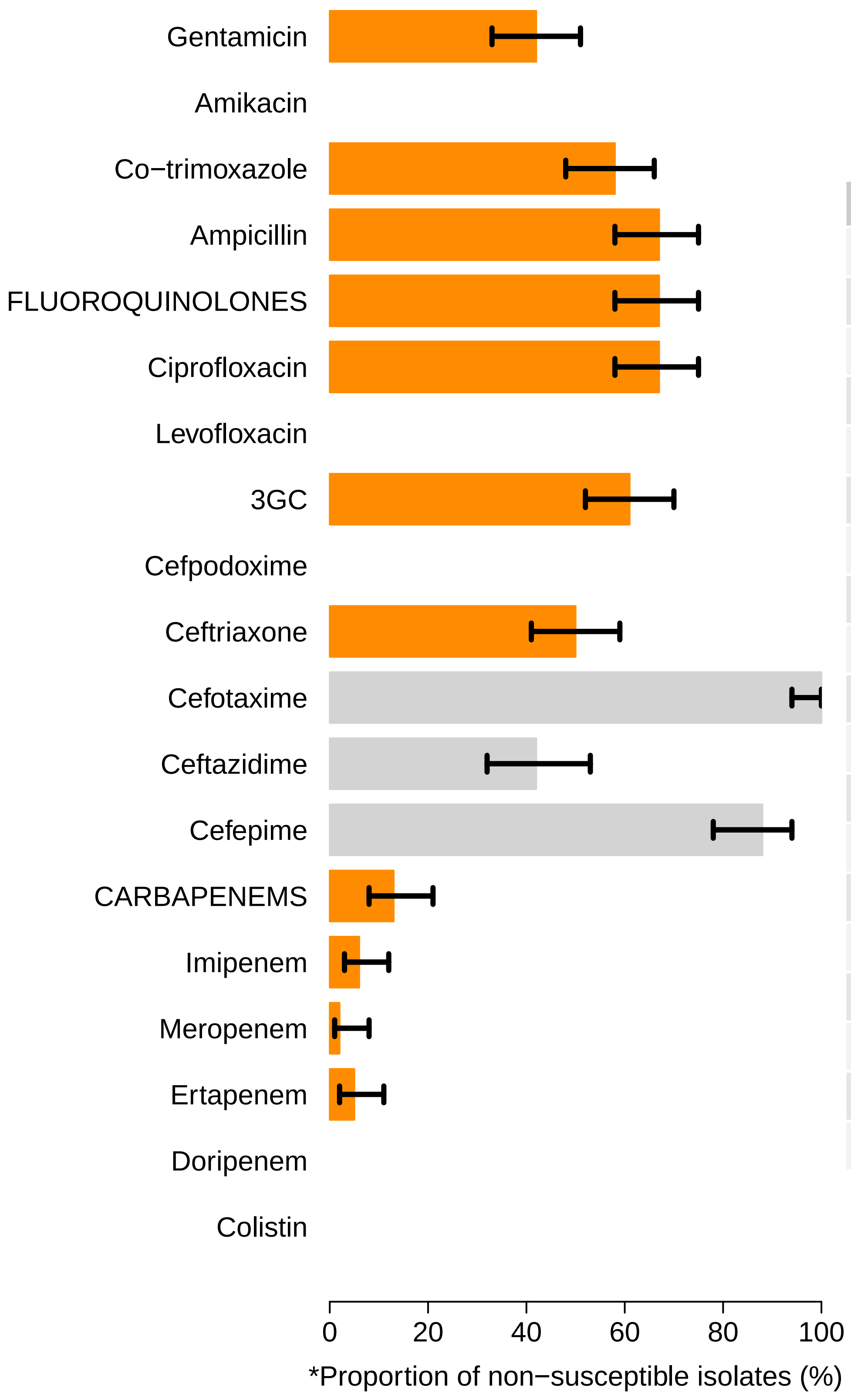

Antibiotic agent	% NS (n)	95% CI
Gentamicin	42% (47/113)	33%−51%
Amikacin	NA	−
Co−trimoxazole	58% (65/113)	48%−66%
Ampicillin	67% (76/113)	58%−75%
FLUOROQUINOLONES	67% (76/113)	58%−75%
Ciprofloxacin	67% (76/113)	58%−75%
Levofloxacin	NA	−
3GC	61% (69/113)	52%−70%
Cefpodoxime	NA	−
Ceftriaxone	50% (56/113)	41%−59%
Cefotaxime	100% (56/56)	94%−100%
Ceftazidime	42% (32/76)	32%−53%
Cefepime	88% (57/65)	78%−94%
CARBAPENEMS	13% (15/113)	8%−21%
Imipenem	6% (7/113)	3%−12%
Meropenem	2% (2/89)	1%−8%
Ertapenem	5% (6/113)	2%−11%
Doripenem	NA	−
Colistin	NA	−

*Proportion of non−susceptible isolates (% NS) represents the number of patients with blood culture positive for non−susceptible isolates (numerator) over the total number of patients with blood culture positive for the organism and the organism was tested for susceptibility against the antibiotic (denominator). The AMASS application de−duplicated the data by including only the first isolate per patient per specimen type per evaluation period. Grey bars indicate that testing with the antibiotic occurred for less than 70% of the total number of blood culture positive for the organism.
CI = confidence interval; NA = Not available/reported/tested; 3GC = 3rd−generation cephalosporin; FLUOROQUINOLONES: ciprofloxacin or levofloxacin; CARBAPENEMS: imipenem, meropenem, ertapenem or doripenem

Section [3]: Isolate−based surveillance report with stratification by infection origin

Blood: ***Klebsiella pneumoniae*** **Community−origin** ***(No. of patients = 140)***

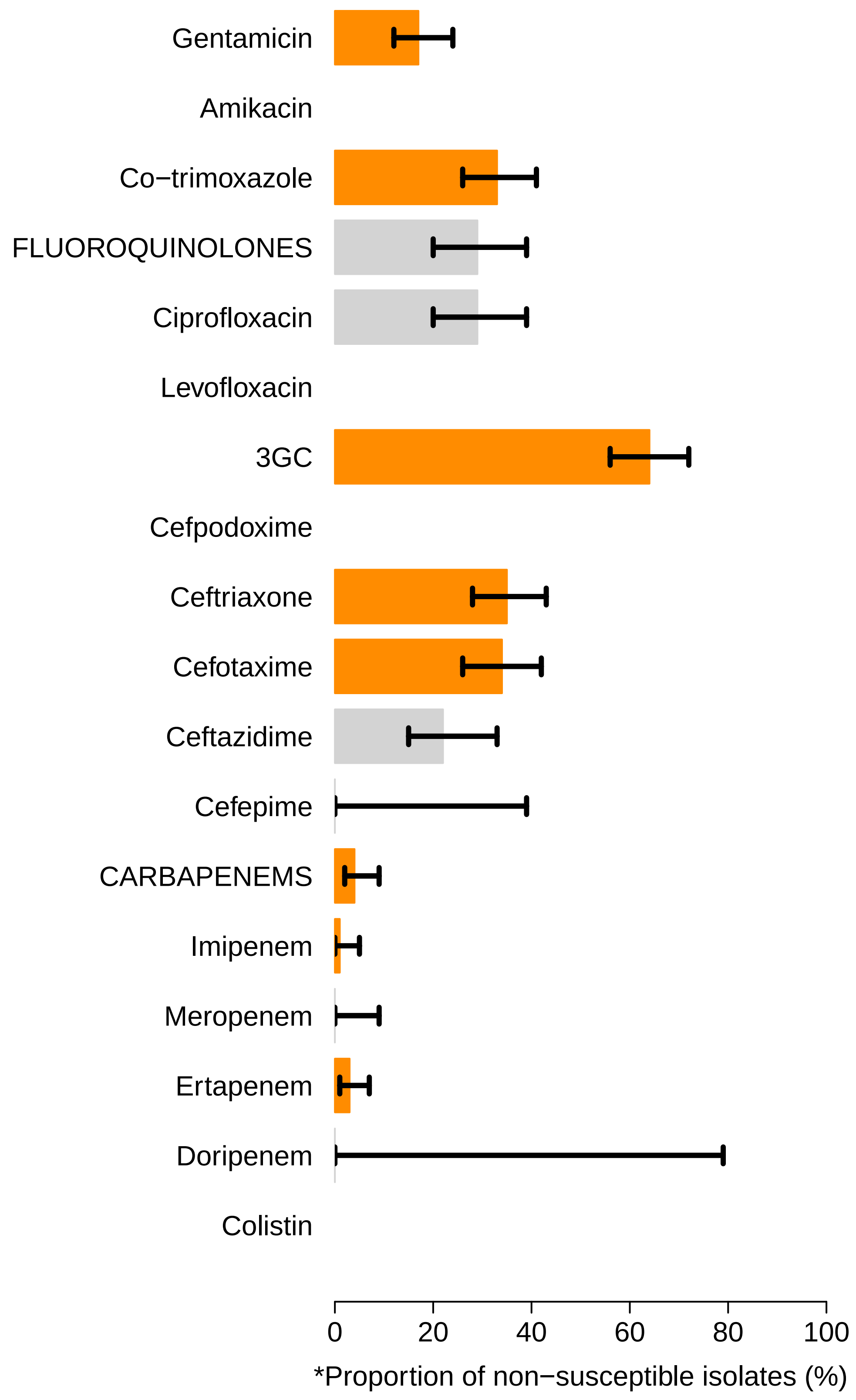

Antibiotic agent	% NS (n)	95% CI
Gentamicin	17% (24/140)	12%−24%
Amikacin	NA	−
Co−trimoxazole	33% (46/140)	26%−41%
FLUOROQUINOLONES	29% (26/91)	20%−39%
Ciprofloxacin	29% (26/91)	20%−39%
Levofloxacin	NA	−
3GC	64% (90/140)	56%−72%
Cefpodoxime	NA	−
Ceftriaxone	35% (49/140)	28%−43%
Cefotaxime	34% (47/140)	26%−42%
Ceftazidime	22% (18/80)	15%−33%
Cefepime	0% (0/6)	0%−39%
CARBAPENEMS	4% (6/140)	2%−9%
Imipenem	1% (2/140)	0%−5%
Meropenem	0% (0/40)	0%−9%
Ertapenem	3% (4/140)	1%−7%
Doripenem	0% (0/1)	0%−79%
Colistin	NA	−

*Proportion of non−susceptible isolates (% NS) represents the number of patients with blood culture positive for non−susceptible isolates (numerator) over the total number of patients with blood culture positive for the organism and the organism was tested for susceptibility against the antibiotic (denominator). The AMASS application de−duplicated the data by including only the first isolate per patient per specimen type per evaluation period. Grey bars indicate that testing with the antibiotic occurred for less than 70% of the total number of blood culture positive for the organism.
CI = confidence interval; NA = Not available/reported/tested; 3GC = 3rd−generation cephalosporin; FLUOROQUINOLONES: ciprofloxacin or levofloxacin; CARBAPENEMS: imipenem, meropenem, ertapenem or doripenem

Section [3]: Isolate−based surveillance report with stratification by infection origin

Blood: ***Klebsiella pneumoniae*** **Hospital−origin** ***(No. of patients = 124)***

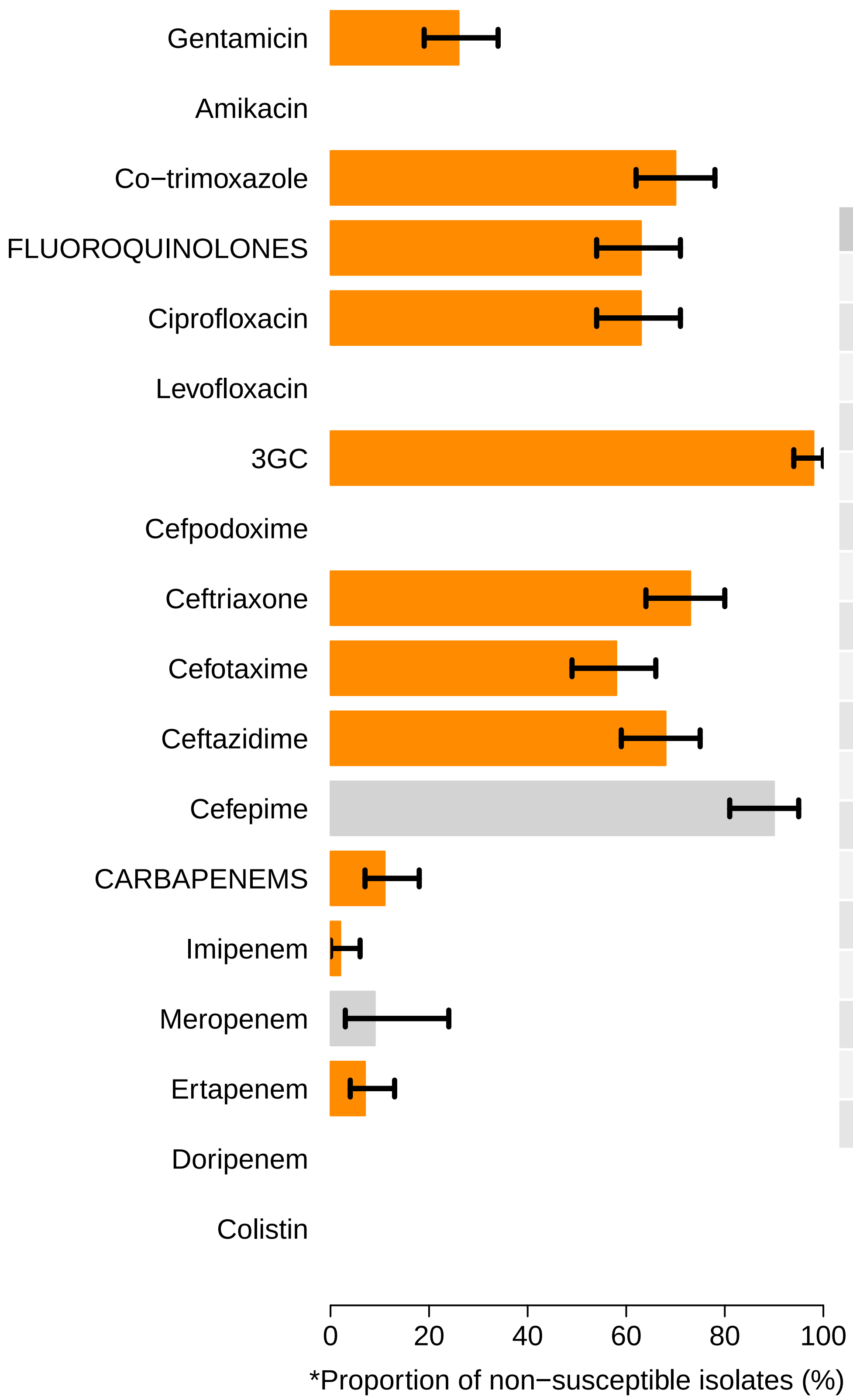

Antibiotic agent	% NS (n)	95% CI
Gentamicin	26% (32/124)	19%−34%
Amikacin	NA	−
Co−trimoxazole	70% (87/124)	62%−78%
FLUOROQUINOLONES	63% (78/124)	54%−71%
Ciprofloxacin	63% (78/124)	54%−71%
Levofloxacin	NA	−
3GC	98% (122/124)	94%−100%
Cefpodoxime	NA	−
Ceftriaxone	73% (90/124)	64%−80%
Cefotaxime	58% (72/124)	49%−66%
Ceftazidime	68% (84/124)	59%−75%
Cefepime	90% (72/80)	81%−95%
CARBAPENEMS	11% (14/124)	7%−18%
Imipenem	2% (2/124)	0%−6%
Meropenem	9% (3/33)	3%−24%
Ertapenem	7% (9/124)	4%−13%
Doripenem	NA	−
Colistin	NA	−

*Proportion of non−susceptible isolates (% NS) represents the number of patients with blood culture positive for non−susceptible isolates (numerator) over the total number of patients with blood culture positive for the organism and the organism was tested for susceptibility against the antibiotic (denominator). The AMASS application de−duplicated the data by including only the first isolate per patient per specimen type per evaluation period. Grey bars indicate that testing with the antibiotic occurred for less than 70% of the total number of blood culture positive for the organism.
CI = confidence interval; NA = Not available/reported/tested; 3GC = 3rd−generation cephalosporin; FLUOROQUINOLONES: ciprofloxacin or levofloxacin; CARBAPENEMS: imipenem, meropenem, ertapenem or doripenem

Section [3]: Isolate−based surveillance report with stratification by infection origin

Blood: *Pseudomonas aeruginosa* Community−origin ***(No. of patients = 35)***

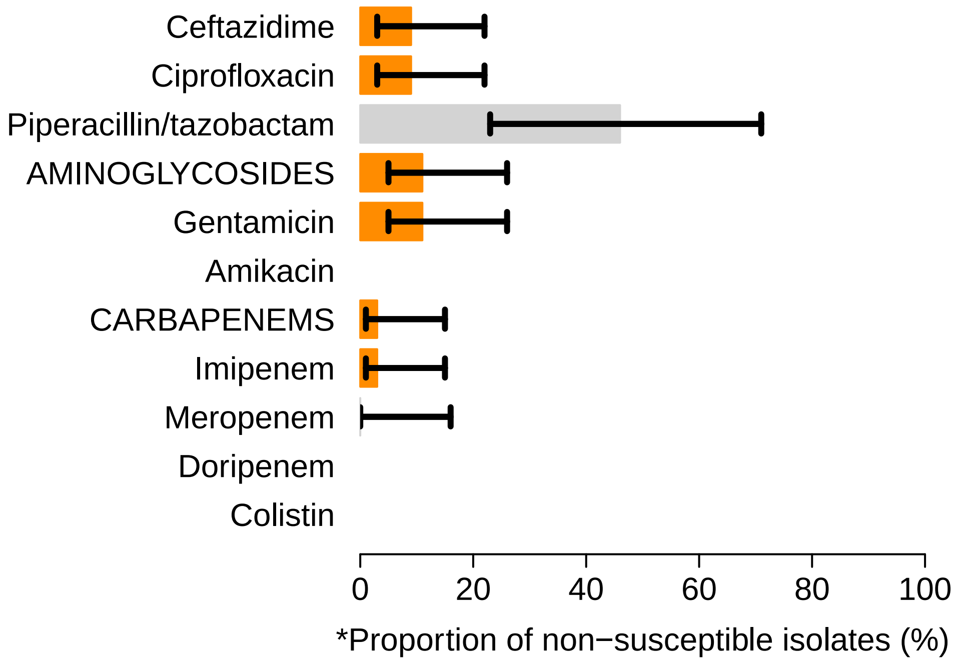

Antibiotic agent	% NS (n)	95% CI
Ceftazidime	9% (3/35)	3%−22%
Ciprofloxacin	9% (3/35)	3%−22%
Piperacillin/tazobactam	46% (6/13)	23%−71%
AMINOGLYCOSIDES	11% (4/35)	5%−26%
Gentamicin	11% (4/35)	5%−26%
Amikacin	NA	−
CARBAPENEMS	3% (1/35)	1%−15%
Imipenem	3% (1/35)	1%−15%
Meropenem	0% (0/20)	0%−16%
Doripenem	NA	−
Colistin	NA	−

Blood: *Pseudomonas aeruginosa* Hospital−origin ***(No. of patients = 73)***

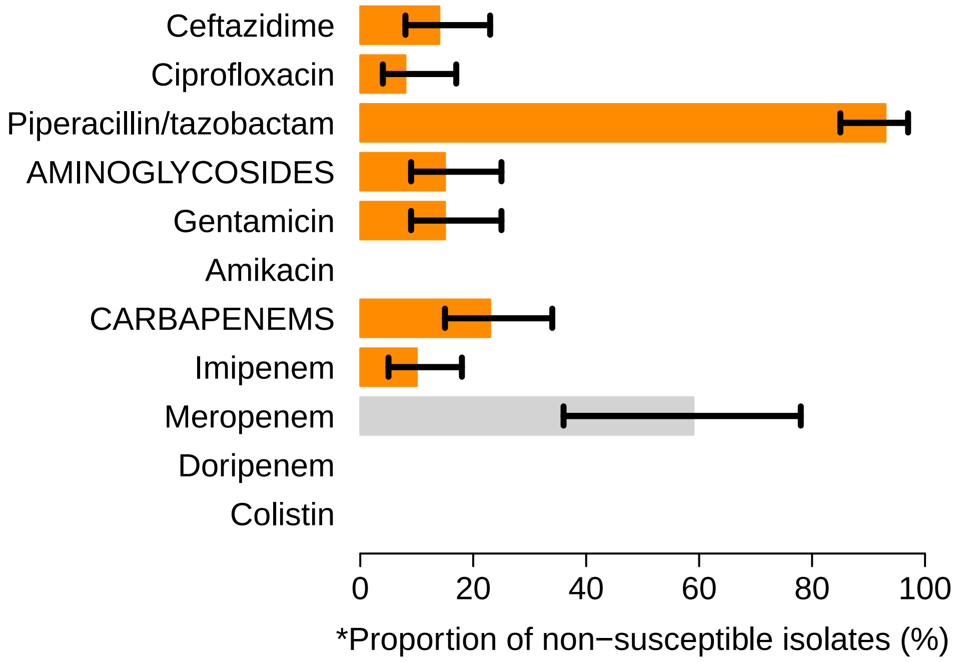

Antibiotic agent	% NS (n)	95% CI
Ceftazidime	14% (10/73)	8%−23%
Ciprofloxacin	8% (6/73)	4%−17%
Piperacillin/tazobactam	93% (68/73)	85%−97%
AMINOGLYCOSIDES	15% (11/73)	9%−25%
Gentamicin	15% (11/73)	9%−25%
Amikacin	NA	−
CARBAPENEMS	23% (17/73)	15%−34%
Imipenem	10% (7/73)	5%−18%
Meropenem	59% (10/17)	36%−78%
Doripenem	NA	−
Colistin	NA	−

*Proportion of non−susceptible isolates (% NS) represents the number of patients with blood culture positive for non−susceptible isolates (numerator) over the total number of patients with blood culture positive for the organism and the organism was tested for susceptibility against the antibiotic (denominator). The AMASS application de−duplicated the data by including only the first isolate per patient per specimen type per evaluation period. Grey bars indicate that testing with the antibiotic occurred for less than 70% of the total number of blood culture positive for the organism.
CI = confidence interval; NA = Not available/reported/tested; AMINOGLYCOSIDES: either gentamicin or amikacin; CARBAPENEMS: imipenem, meropenem, ertapenem or doripenem

Section [3]: Isolate−based surveillance report with stratification by infection origin

Blood: *Acinetobacter* spp. Community−origin (No. of patients = 61)

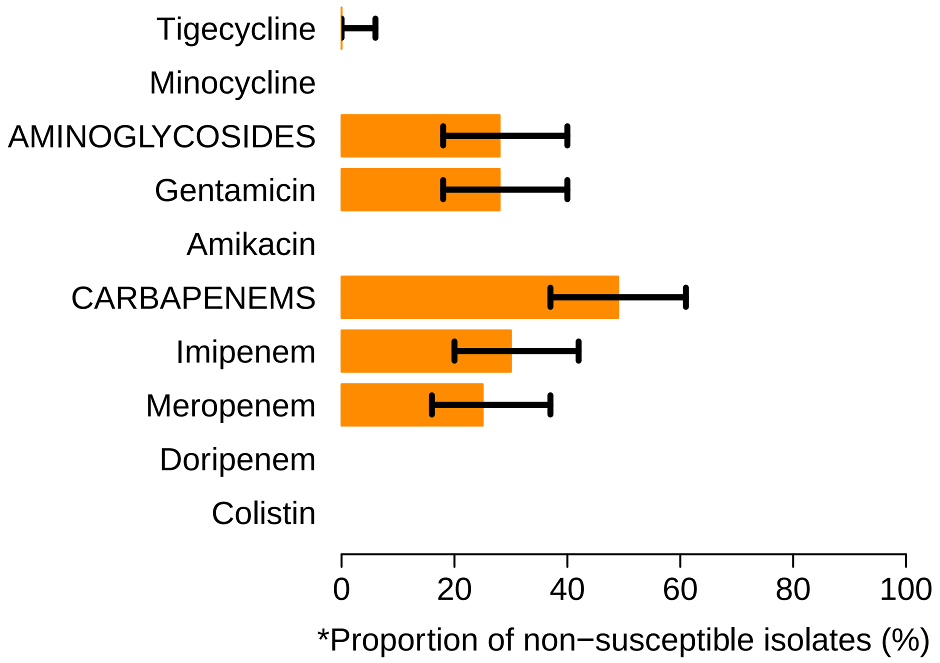

Antibiotic agent	% NS (n)	95% CI
Tigecycline	0% (0/61)	0%−6%
Minocycline	NA	−
AMINOGLYCOSIDES	28% (17/61)	18%−40%
Gentamicin	28% (17/61)	18%−40%
Amikacin	NA	−
CARBAPENEMS	49% (30/61)	37%−61%
Imipenem	30% (18/61)	20%−42%
Meropenem	25% (15/61)	16%−37%
Doripenem	NA	−
Colistin	NA	−

Blood: *Acinetobacter* spp. Hospital−origin (No. of patients = 172)

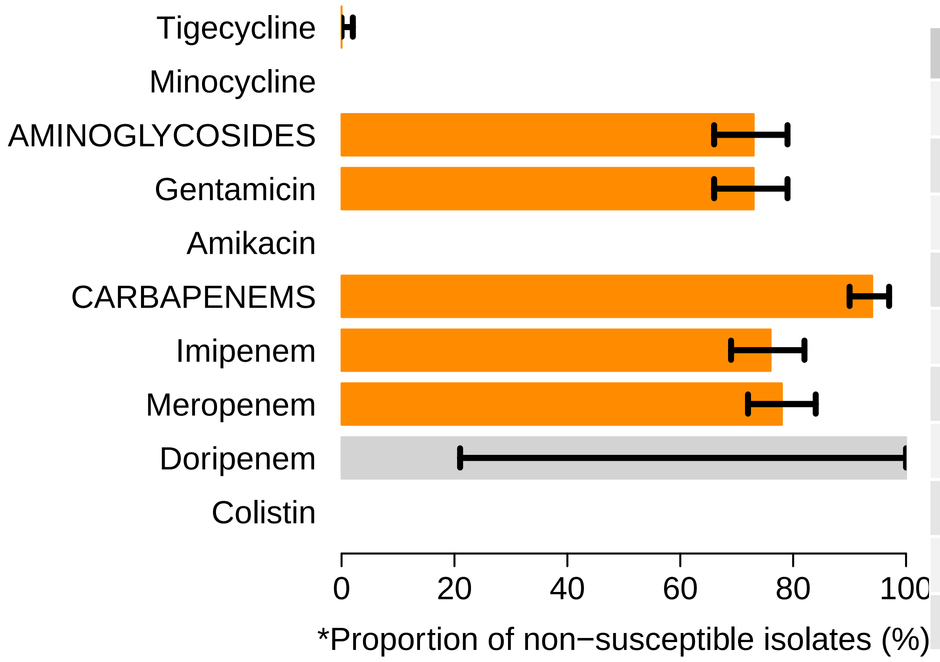

Antibiotic agent	% NS (n)	95% CI
Tigecycline	0% (0/172)	0%−2%
Minocycline	NA	−
AMINOGLYCOSIDES	73% (126/172)	66%−79%
Gentamicin	73% (126/172)	66%−79%
Amikacin	NA	−
CARBAPENEMS	94% (162/172)	90%−97%
Imipenem	76% (131/172)	69%−82%
Meropenem	78% (135/172)	72%−84%
Doripenem	100% (1/1)	21%−100%
Colistin	NA	−

*Proportion of non−susceptible isolates (% NS) represents the number of patients with blood culture positive for non−susceptible isolates (numerator) over the total number of patients with blood culture positive for the organism and the organism was tested for susceptibility against the antibiotic (denominator). The AMASS application de−duplicated the data by including only the first isolate per patient per specimen type per evaluation period. Grey bars indicate that testing with the antibiotic occurred for less than 70% of the total number of blood culture positive for the organism.
CI = confidence interval; NA = Not available/reported/tested; AMINOGLYCOSIDES: either gentamicin or amikacin; CARBAPENEMS: imipenem, meropenem, ertapenem or doripenem

Section [4]: Sample−based surveillance report

Introduction
A sample−based surveillance report is generated if data of culture negative is available.

The sample−based approach involves the collection of data on all blood samples taken for microbiological testing and includes information on the number of positive blood samples for a specific specimen type (both pathogens under the survey and other bacteria) as well as number of negative (no microbial growth) samples. After removal of duplicate results and assuming that routine blood culture testing is applied systematically, we can use the number of tested patients as a proxy for a number of patients with new cases of bloodstream infection (BSI).

Results:
The microbiology_data file had:

Specimen collection dates ranged from ***01 Jan 2016 to 31 Dec 2016***
Number of records on blood specimen collected within the above date range:
56995 blood specimen records
**Number of patients sampled for blood culture within the above date range:*
20154 patients sampled for blood culture

Note:
*Number of patients sampled for blood culture is used as denominator to estimate the frequency of infections per 100,000 tested patients

The following figures show the frequncy of infections for patients with blood culture tested.

Section [4]: Sample−based surveillance report

Blood: ***Pathogens under this surveillance** ***(No. of patients = 20154)***

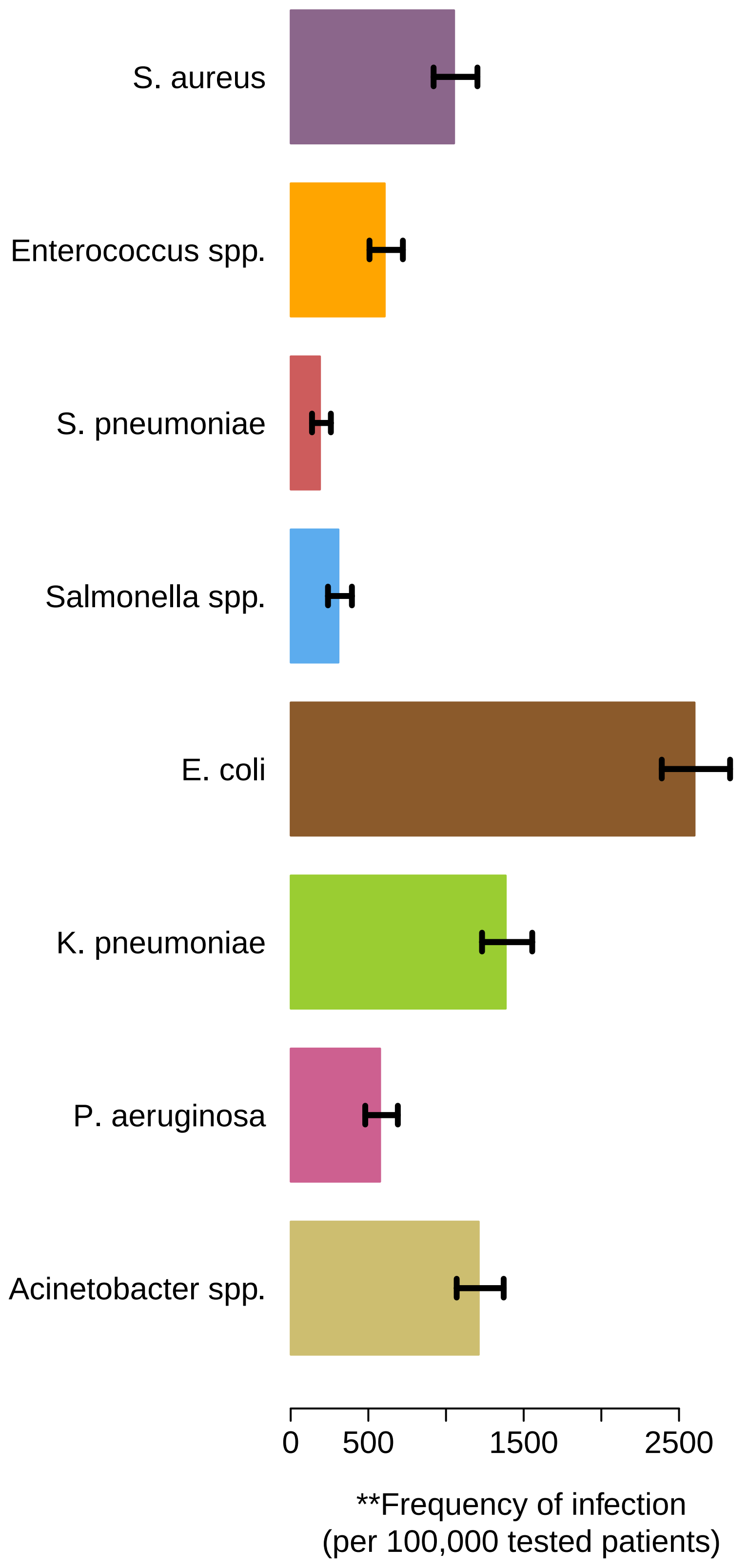

Organisms	**Frequency (95% CI)
S. aureus	1052 (921−1203)
Enterococcus spp.	606 (508−723)
S. pneumoniae	189 (138−259)
Salmonella spp.	308 (241−395)
E. coli	2600 (2390−2829)
K. pneumoniae	1385 (1233−1556)
P. aeruginosa	576 (481−690)
Acinetobacter spp.	1211 (1069−1372)

*We apologise that the bacteria name in the table and in the figure are not written in italic. This is because of the R command we used. We will improve this in the next version.
**Frequency of infection per 100,000 tested patients represents the number of patients with blood culture positive for a pathogen (numerator) over the total number of tested patients (denominator). The AMASS application de−duplicates the data by included only the first isolate of each patient per specimen type per reporting period.
CI = confidence interval; NS = non−susceptible; NA = Not available/reported/tested; 3GC = 3rd−generation cephalosporin

Section [4]: Sample−based surveillance report

Blood: ***AMR pathogens under this surveillance** ***(No. of patients = 20154)***

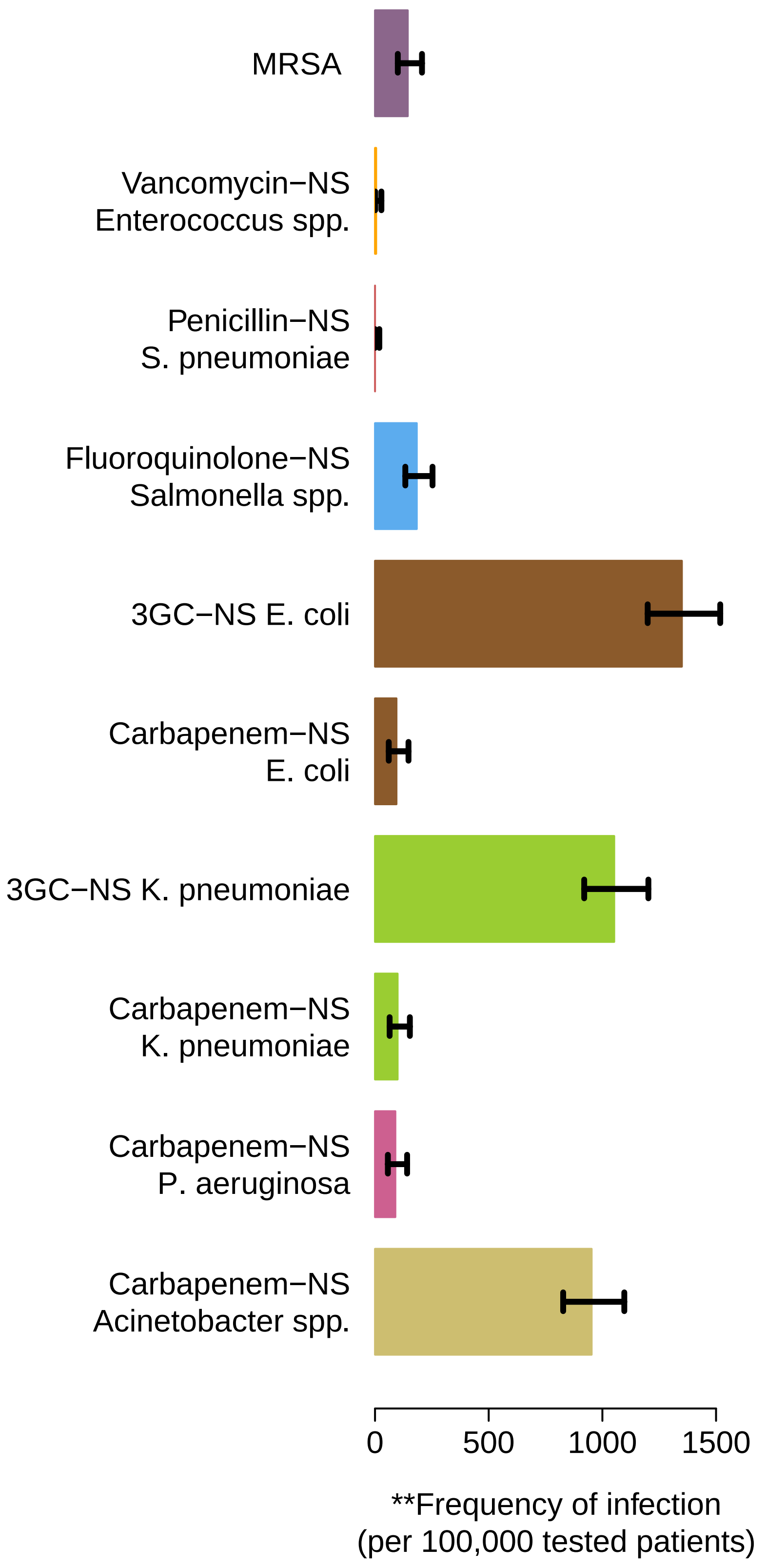

Organism	**Frequency (95% CI)
MRSA	144 (101−207)
Vancomycin−NS Enterococcus spp.	5 (1−29)
Penicillin−NS S. pneumoniae	0 (0−20)
Fluoroquinolone−NS Salmonella spp.	184 (134−253)
3GC−NS E. coli	1350 (1200−1519)
Carbapenem−NS E. coli	95 (61−148)
3GC−NS K. pneumoniae	1052 (921−1203)
Carbapenem−NS K. pneumoniae	100 (65−154)
Carbapenem−NS P. aeruginosa	90 (57−142)
Carbapenem−NS Acinetobacter spp.	953 (828−1097)

*We apologise that the bacteria name in the table and in the figure are not written in italic. This is because of the R command we used. We will improve this in the next version.
**Frequency of infection per 100,000 tested patients represents the number of patients with blood culture positive for a pathogen (numerator) over the total number of tested patients (denominator). The AMASS application de−duplicates the data by included only the first isolate of each patient per specimen type per reporting period.
CI = confidence interval; NS = non−susceptible; NA = Not available/reported/tested; 3GC = 3rd−generation cephalosporin

Section [5]: Sample−based surveillance report with stratification by infection origin

Introduction

A sample−based surveillance report with stratification by origin of infection is generated only if data of culture negative is available and admission date or a variable containing the classification is available in the raw data file with the appropriate specification in the data dictionaries.

Results:

The data included in the analysis had:

Specimen collection dates ranged from ***01 Jan 2016 to 31 Dec 2016***

Number of records on blood specimen collected within the above date range:
56995 blood specimen records

Number of patients sampled for blood culture within the above date range:
20154 patients sampled for blood culture

16064 *patients had at least one admission having the first blood culture drawn within first 2 calendar days of hospital admission.*
This parameter is used as a denominators for frequency of community−origin bacteraemia (per 100,000 patients tested for blood culture on admission).

4082 *patients had at least one admission having the first blood culture drawn after 2 calendar days of hospital admission.*
This parameter is used as a denominators for frequency of hospital−origin bacteraemia (per 100,000 patients tested for blood culture for HAI).

765 *patients had a blood drawn for culture and with unknown origin of infection.*
Validation of this statistics is highly recommended.

Note:

757 *patients had more than one admissions, of which at least one admission had the first blood culture drawn within the first 2 calendar days of hospital admission AND at least one admission had the first blood culture drawn after 2 calendar days of hospital admission.*

The following figures show the frequency of infections for patients with blood culture tested and stratified by infection origin, under this surveillance.

Section [5]: Sample−based surveillance report with stratification by infection origin

Blood: ***Pathogens** **Community−origin** ***(No. of patients = 16064)***

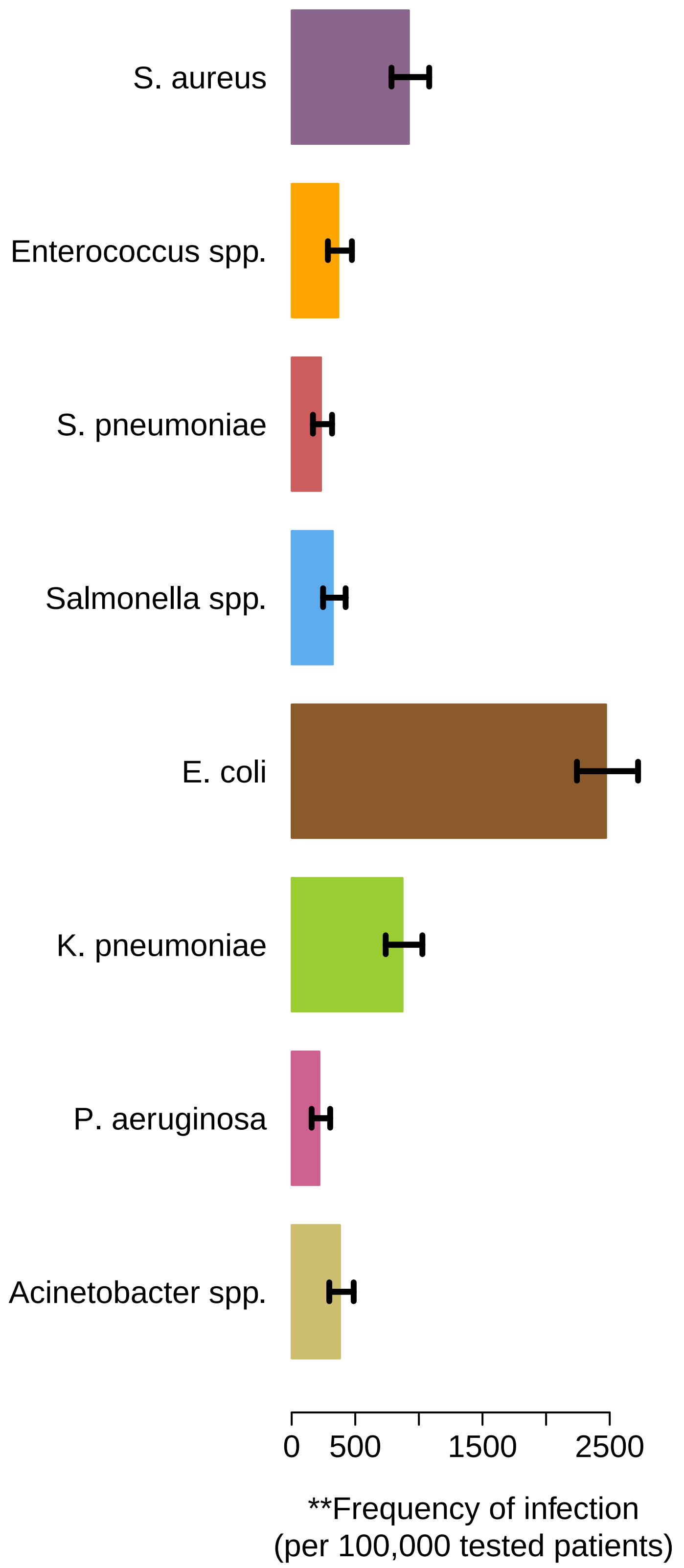

Organism	**Frequency (95% CI)
S. aureus	922 (785−1082)
Enterococcus spp.	368 (285−474)
S. pneumoniae	231 (168−318)
Salmonella spp.	324 (247−425)
E. coli	2472 (2243−2724)
K. pneumoniae	872 (740−1028)
P. aeruginosa	218 (157−303)
Acinetobacter spp.	380 (296−488)

*We apologise that the bacteria name in the table and in the figure are not written in italic. This is because of the R command we used. We will improve this in the next version.
**Frequency of infection per 100,000 tested patients on admission represents the number of patients with blood culture positive for a pathogen (numerator) over the total number of tested population on admission (denominator). The AMASS application de−duplicates the data by included only the first isolate of each patient per specimen type per reporting period.
CI = confidence interval; NS = non−susceptible; NA = Not available/reported/tested; 3GC = 3rd−generation cephalosporin

Section [5]: Sample−based surveillance report with stratification by infection origin

Blood: ***Pathogens** **Hospital−origin** ***(No. of patients = 4082)***

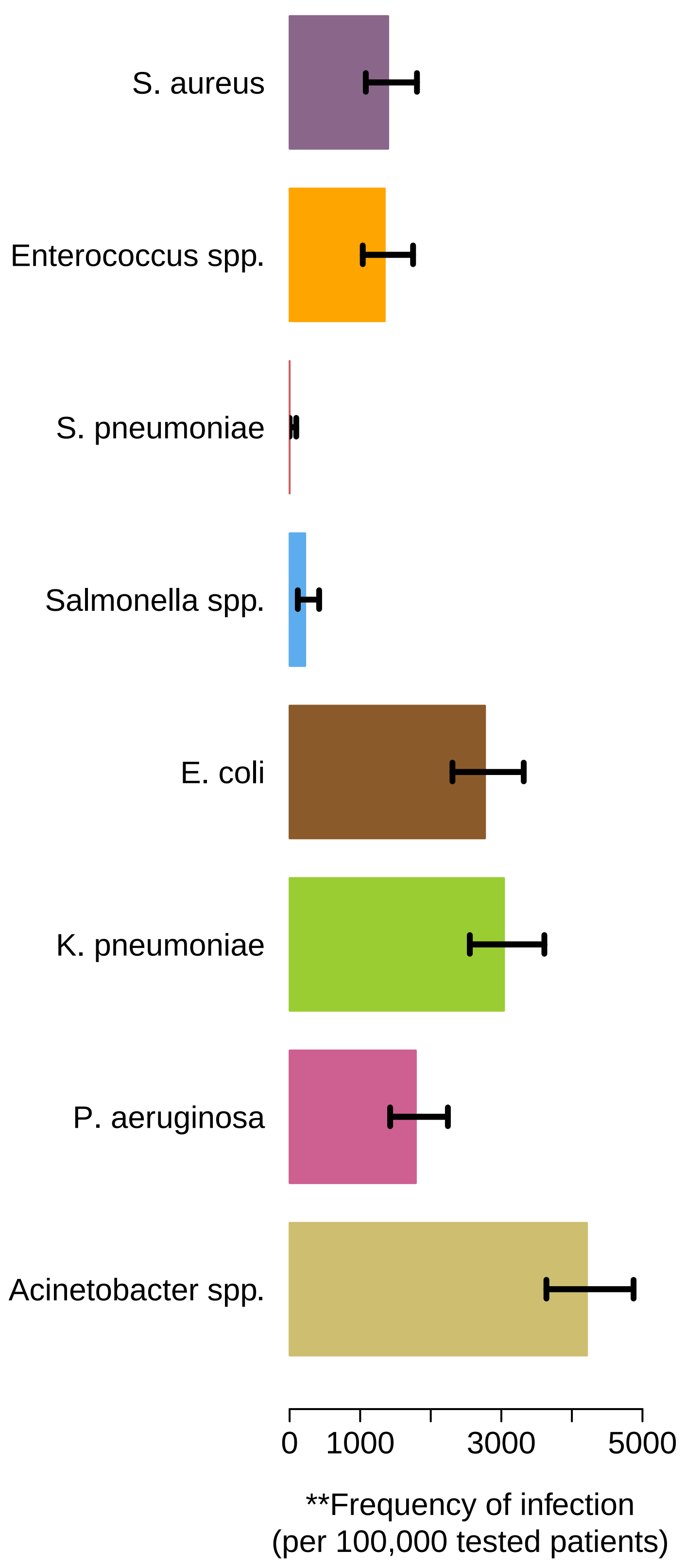

Organism	**Frequency (95% CI)
S. aureus	1397 (1080−1805)
Enterococcus spp.	1348 (1037−1750)
S. pneumoniae	0 (0−95)
Salmonella spp.	221 (117−419)
E. coli	2769 (2308−3318)
K. pneumoniae	3038 (2554−3610)
P. aeruginosa	1789 (1425−2243)
Acinetobacter spp.	4214 (3640−4875)

*We apologise that the bacteria name in the table and in the figure are not written in italic. This is because of the R command we used. We will improve this in the next version.
**Frequency of infection per 100,000 tested population at risk of HAI represents the number of patients with blood culture positive for a pathogen (numerator) over the total number of tested population at risk of HAI (denominator). The AMASS application de−duplicates the data by included only the first isolate of each patient per specimen type per reporting period.
CI = confidence interval; NS = non−susceptible; NA = Not available/reported/tested; 3GC = 3rd−generation cephalosporin

Section [5]: Sample−based surveillance report with stratification by infection origin

Blood: ***AMR pathogens** **Community−origin** ***(No. of patients = 16064)***

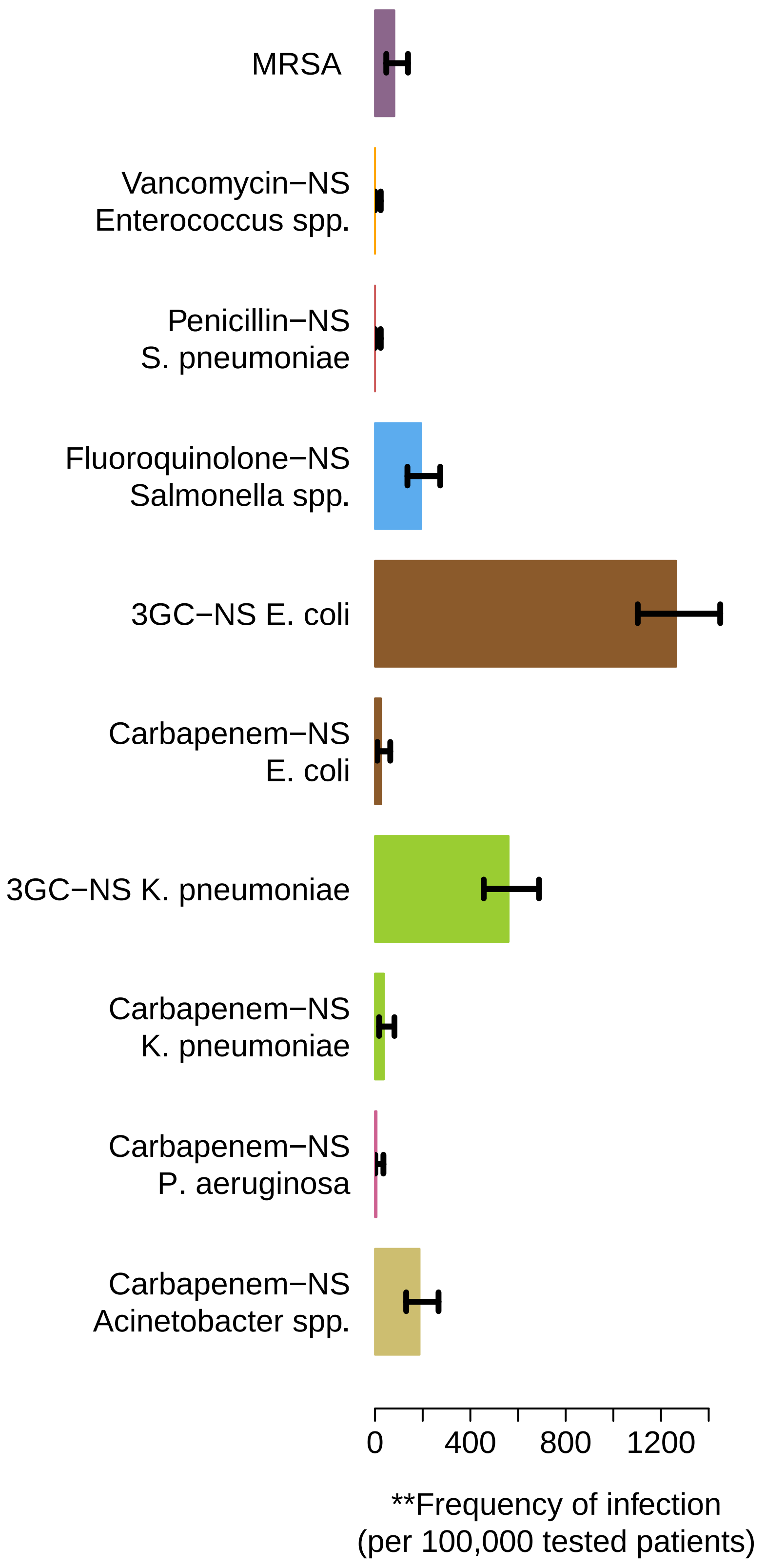

Organism	**Frequency (95% CI)
MRSA	81 (48−139)
Vancomycin−NS Enterococcus spp.	0 (0−24)
Penicillin−NS S. pneumoniae	0 (0−24)
Fluoroquinolone−NS Salmonella spp.	193 (136−274)
3GC−NS E. coli	1264 (1103−1449)
Carbapenem−NS E. coli	25 (10−65)
3GC−NS K. pneumoniae	561 (457−689)
Carbapenem−NS K. pneumoniae	38 (18−82)
Carbapenem−NS P. aeruginosa	7 (2−36)
Carbapenem−NS Acinetobacter spp.	187 (131−267)

*We apologise that the bacteria name in the table and in the figure are not written in italic. This is because of the R command we used. We will improve this in the next version.
**Frequency of infection per 100,000 tested patients represents the number of patients with blood culture positive for a pathogen (numerator) over the total number of tested patients (denominator). The AMASS application de−duplicates the data by included only the first isolate of each patient per specimen type per reporting period.
CI = confidence interval; NS = non−susceptible; NA = Not available/reported/tested; 3GC = 3rd−generation cephalosporin

Section [5]: Sample−based surveillance report with stratification by infection origin

Blood: ***AMR pathogens** **Hospital−origin** ***(No. of patients = 4082)***

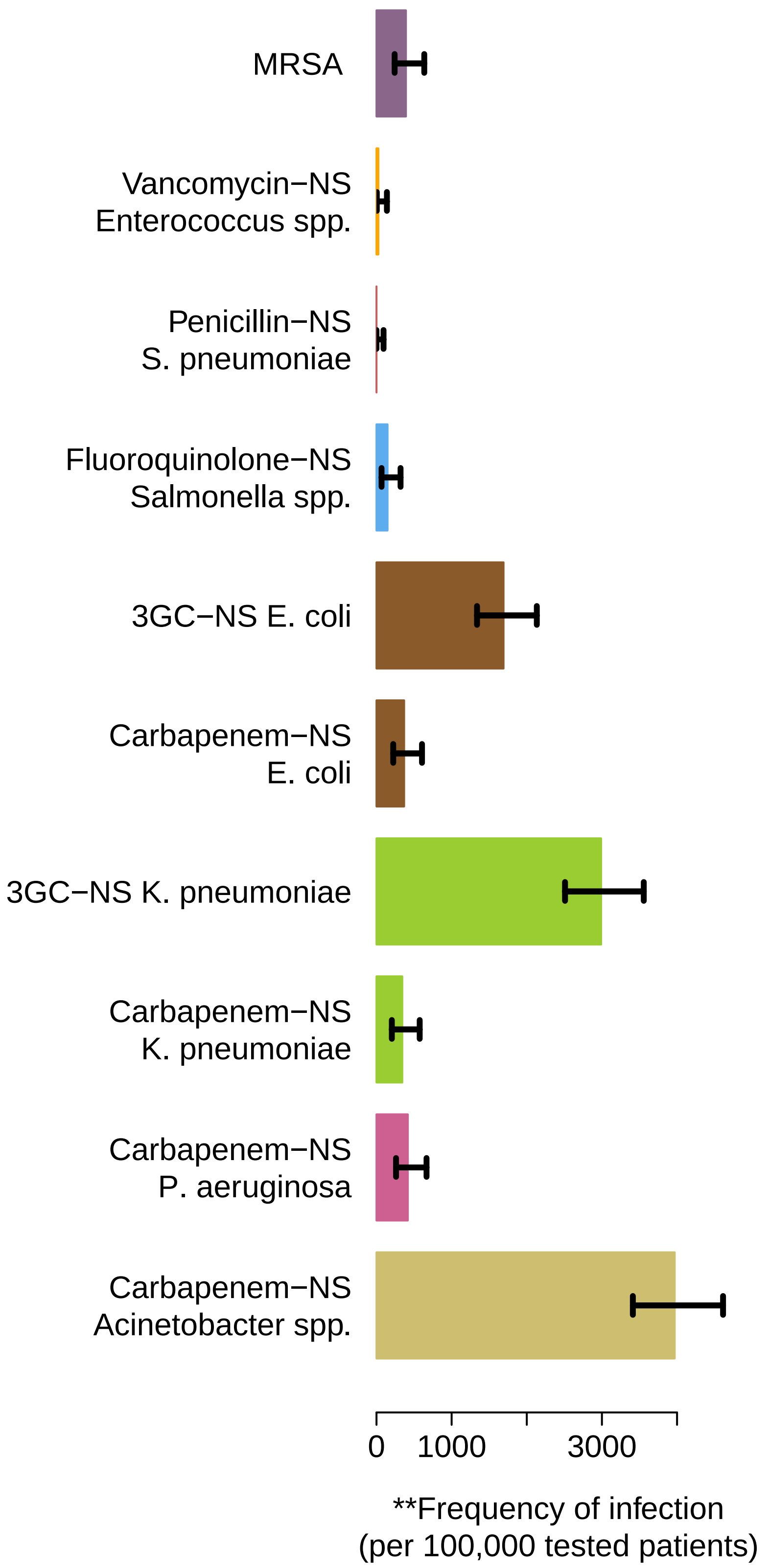

Organism	**Frequency (95% CI)
MRSA	392 (242−636)
Vancomycin−NS Enterococcus spp.	25 (5−139)
Penicillin−NS S. pneumoniae	0 (0−95)
Fluoroquinolone−NS Salmonella spp.	147 (68−321)
3GC−NS E. coli	1691 (1338−2134)
Carbapenem−NS E. coli	368 (223−606)
3GC−NS K. pneumoniae	2989 (2509−3557)
Carbapenem−NS K. pneumoniae	343 (205−575)
Carbapenem−NS P. aeruginosa	417 (261−666)
Carbapenem−NS Acinetobacter spp.	3969 (3412−4613)

*We apologise that the bacteria name in the table and in the figure are not written in italic. This is because of the R command we used. We will improve this in the next version.
**Frequency of infection per 100,000 tested patients represents the number of patients with blood culture positive for a pathogen (numerator) over the total number of tested patients (denominator). The AMASS application de−duplicates the data by included only the first isolate of each patient per specimen type per reporting period.
CI = confidence interval; NS = non−susceptible; NA = Not available/reported/tested; 3GC = 3rd−generation cephalosporin

Section [6] Mortality involving AMR and antimicrobial−susceptible infections

Introduction

A surveillance report on mortality involving AMR infections and antimicrobial−susceptible infections with stratification by origin of infection is generated only if data on patient outcomes (i.e. discharge status) are available. Antimicrobial−resistant infection is a threat tc modern health care, and the impact of the infection on patient outcomes is largely unknown Performing analyses and generating reports on mortality often takes time and resources.

The term 'mortality involving AMR and antimicrobial−susceptible infections was used because the mortality reported was all−cause mortality. This measure of mortality included deaths caused by or related to other underlying and intermediate causes.

Here, AMASS summarized the overall mortality of patients with antimicrobial−resistant and antimicrobial−susceptible bacteria bloodstream infections (BSI).

Results:

The data included in the analysis had:

Sample collection dates ranged from ***01 Jan 2016*** to ***31 Dec 2016***

Number of patients with blood culture positive for the origanism under the survey:

1597 patients

Number of patients with community−origin BSI:

929 patients

Number of patients with hospital−origin BSI:

603 patients

The hospital admission data file had:

Hospital admission dates ranging from ***01 Jan 2016*** to ***31 Dec 2016***

Number of records in the raw hospital admission data:

109450 records

Number of patients included in the analysis (de−duplicated):

20194 patients

Number of patients had death as outcome in any admission data records:

1314 patients

Overall mortality:

7% (1314/20194)

The AMASS application merged the microbiology data file and hospital admission data file. The merged dataset was then de−duplicated so that only the first isolate per patient per specimen per reporting period was included in the analysis. The de−duplicated data was stratified by infection origin (community−origin infection or hospital−origin infection).

**Organism**	**Mortality in patients with Community−origin BSI**	**Mortality in patients with Hospital−origin BSI**
Staphylococcus aureus	***11% (17/148)***	***11% (6/57)***
***Enterococcus* spp.**	***8% (5/59)***	***25% (14/55)***
Streptococcus pneumoniae	***8% (3/37)***	***NaN% (0/0)***
***Salmonella* spp.**	***13% (7/52)***	***0% (0/9)***
Escherichia coli	***17% (67/397)***	***17% (19/113)***
Klebsiella pneumoniae	***15% (21/140)***	***23% (29/124)***
Pseudomonas aeruginosa	***26% (9/35)***	***30% (22/73)***
***Acinetobacter* spp.**	***28% (17/61)***	***35% (60/172)***
**Total:**	***16% (146/929)***	***25% (150/603)***

The following figures and tables show the mortality of patients who were blood culture positive for antimicrobial non−susceptible and susceptible isolates.

Section [6] Mortality involving AMR and antimicrobial−susceptible infections

Staphylococcus aureus Community−origin *(No. of patients = 148)*

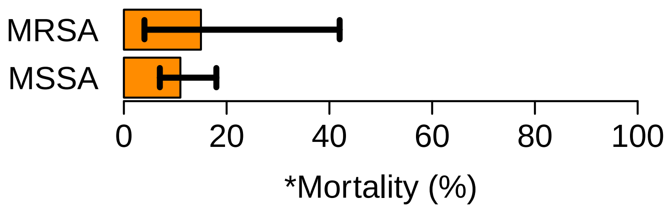

Type of pathogen	Mortality (n)	95% CI
MRSA	15% (2/13)	4%−42%
MSSA	11% (15/135)	7%−18%

Staphylococcus aureus Hospital−origin *(No. of patients = 57)*

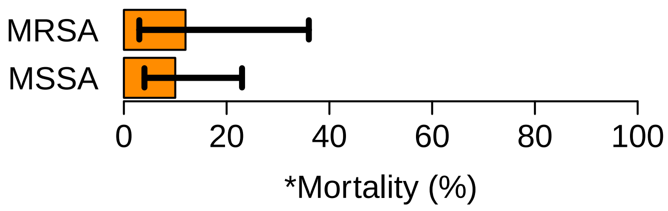

Type of pathogen	Mortality (n)	95% CI
MRSA	12% (2/16)	3%−36%
MSSA	10% (4/41)	4%−23%

Enterococcus spp. Community−origin *(No. of patients = 59)*

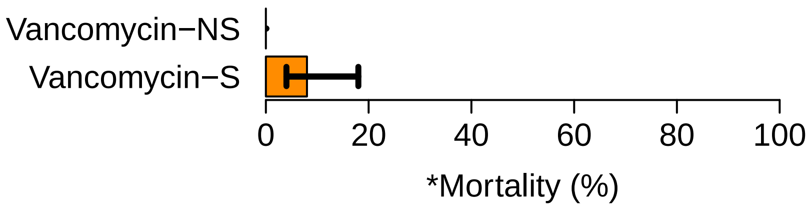

Type of pathogen	Mortality (n)	95% CI
Vancomycin−NS	NA	−
Vancomycin−S	8% (5/59)	4%−18%

Enterococcus spp. Hospital−origin *(No. of patients = 55)*

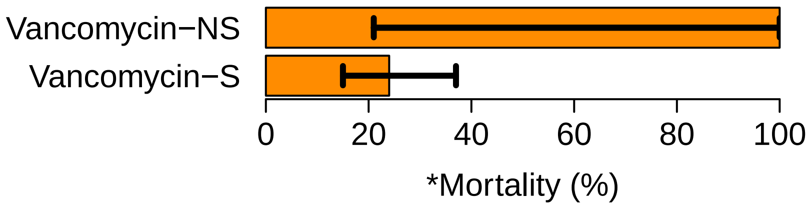

Type of pathogen	Mortality (n)	95% CI
Vancomycin−NS	100% (1/1)	21%−100%
Vancomycin−S	24% (13/54)	15%−37%

*Mortality is the proportion (%) of in−hospital deaths (all−cause deaths). This represents the number of in−hospital deaths (numerator) over the total number of patients with blood culture positive for the organism and the type of pathogen (denominator). The AMASS application de−duplicates the data by included only the first isolate per patient per specimen type per evaluation period. NS = non−susceptible; S = susceptible; CI = confidence interval; Fluoroquinolone−NS = NS to any fluoroquinolone tested; 3GC−NS = NS to any 3rd−generation cephalosporin and susceptible to carbapenem

Section [6] Mortality involving AMR and antimicrobial−susceptible infections

Streptococcus pneumoniae Community−origin ***(No. of patients = 37)***

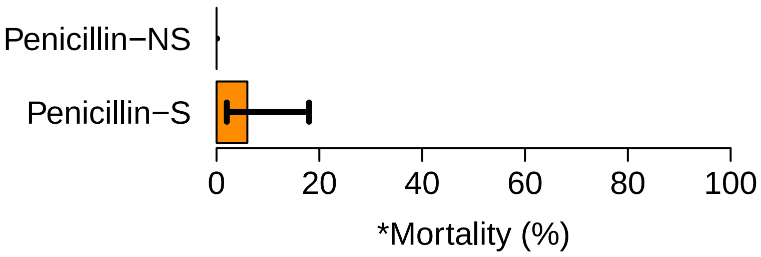

Mortality	Mortality (n)	95% CI
Penicillin−NS	NA	−
Penicillin−S	6% (2/36)	2%−18%

Streptococcus pneumoniae Hospital−origin ***(No. of patients = 0)***

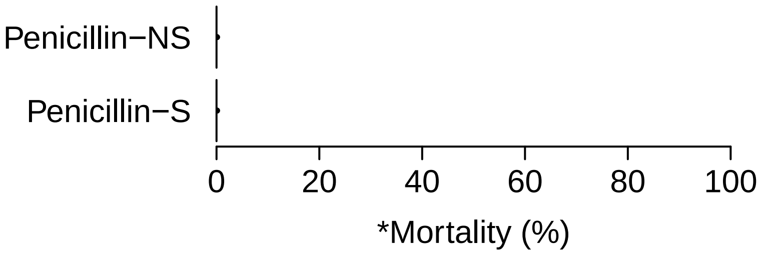

Type of pathogen	Mortality (n)	95% CI
Penicillin−NS	NA	−
Penicillin−S	NA	−

Salmonella spp. Community−origin ***(No. of patients = 52)***

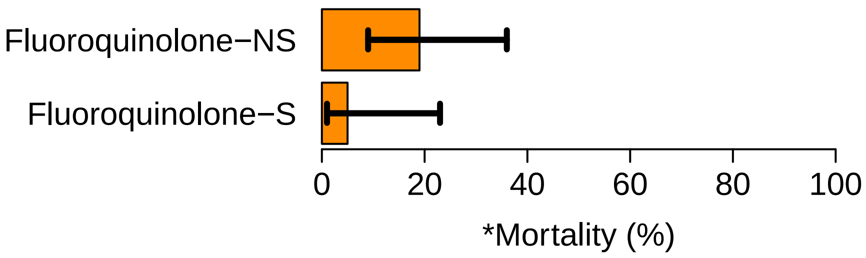

Type of pathogen	Mortality (n)	95% CI
Fluoroquinolone−NS	19% (6/31)	9%−36%
Fluoroquinolone−S	5% (1/21)	1%−23%

Salmonella spp. Hospital−origin ***(No. of patients = 9)***

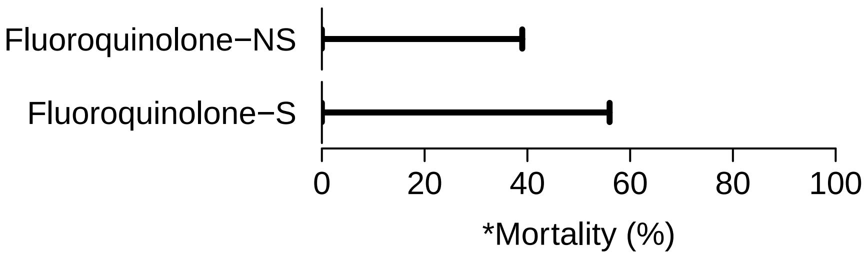

Type of pathogen	Mortality (n)	95% CI
Fluoroquinolone−NS	0% (0/6)	0%−39%
Fluoroquinolone−S	0% (0/3)	0%−56%

*Mortality is the proportion (%) of in−hospital deaths (all−cause deaths). This represents the number of in−hospital deaths (numerator) over the total number of patients with blood culture positive for the organism and the type of pathogen (denominator). The AMASS application de−duplicates the data by included only the first isolate per patient per specimen type per evaluation period. NS = non−susceptible; S = susceptible; CI = confidence interval; Fluoroquinolone−NS = NS to any fluoroquinolone tested; 3GC−NS = NS to any 3rd−generation cephalosporin and susceptible to carbapenem

Section [6] Mortality involving AMR and antimicrobial−susceptible infections

Escherichia coli — Community−origin ***(No. of patients = 397)***

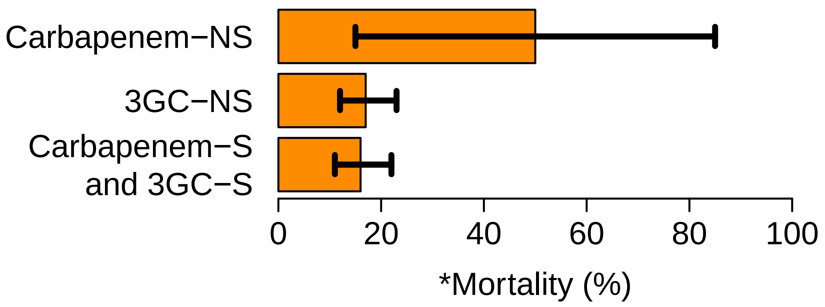

Type of pathogen	Mortality (n)	95% CI
Carbapenem−NS	50% (2/4)	15%−85%
3GC−NS	17% (34/199)	12%−23%
Carbapenem−S and 3GC−S	16% (31/194)	11%−22%

Escherichia coli — Hospital−origin ***(No. of patients = 113)***

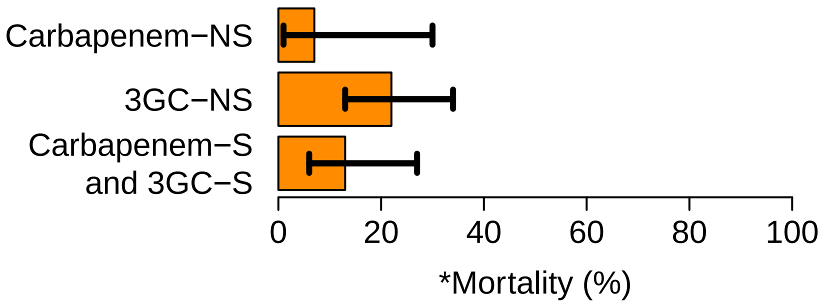

Type of pathogen	Mortality (n)	95% CI
Carbapenem−NS	7% (1/15)	1%−30%
3GC−NS	22% (13/59)	13%−34%
Carbapenem−S and 3GC−S	13% (5/39)	6%−27%

Klebsiella pneumoniae — Community−origin ***(No. of patients = 140)***

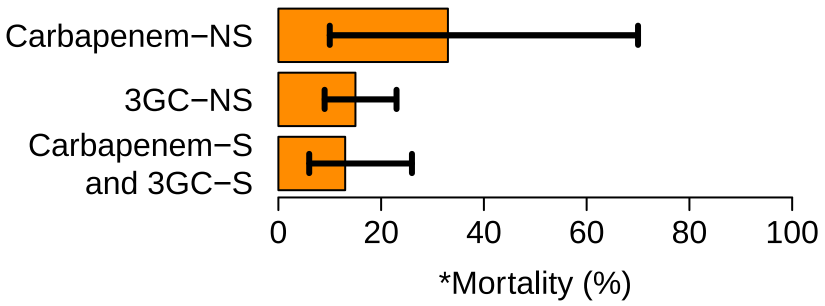

Type of pathogen	Mortality (n)	95% CI
Carbapenem−NS	33% (2/6)	10%−70%
3GC−NS	15% (13/89)	9%−23%
Carbapenem−S and 3GC−S	13% (6/45)	6%−26%

Klebsiella pneumoniae — Hospital−origin ***(No. of patients = 124)***

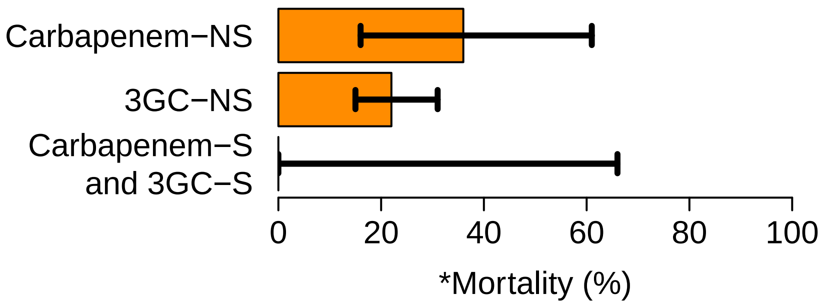

Type of pathogen	Mortality (n)	95% CI
Carbapenem−NS	36% (5/14)	16%−61%
3GC−NS	22% (24/108)	15%−31%
Carbapenem−S and 3GC−S	0% (0/2)	0%−66%

*Mortality is the proportion (%) of in−hospital deaths (all−cause deaths). This represents the number of in−hospital deaths (numerator) over the total number of patients with blood culture positive for the organism and the type of pathogen (denominator). The AMASS application de−duplicates the data by including only the first isolate per patient per specimen type per evaluation period. NS = non−susceptible; S = susceptible; CI = confidence interval; Carbapenem−NS = NS to any carbapenems tested; 3GC−NS = NS to any 3rd−generation cephalosporin and susceptible to carbapenem

Section [6] Mortality involving AMR and antimicrobial−susceptible infections

Pseudomonas aeruginosa — Community−origin *(No. of patients = 35)*

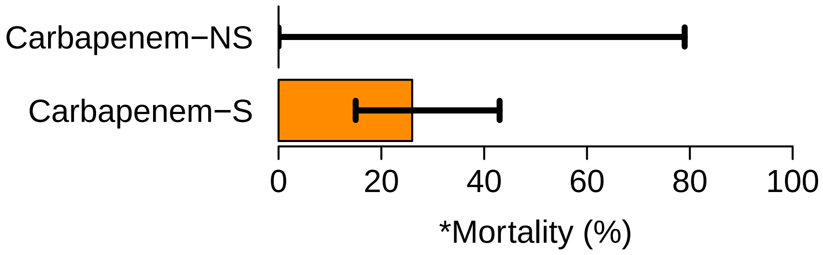

Type of pathogen	Mortality (n)	95% CI
Carbapenem−NS	0% (0/1)	0%−79%
Carbapenem−S	26% (9/34)	15%−43%

Pseudomonas aeruginosa — Hospital−origin *(No. of patients = 73)*

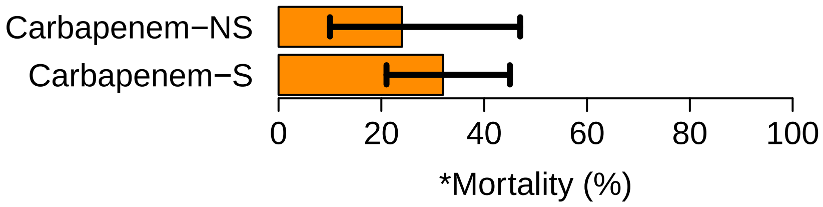

Type of pathogen	Mortality (n)	95% CI
Carbapenem−NS	24% (4/17)	10%−47%
Carbapenem−S	32% (18/56)	21%−45%

Acinetobacter spp. — Community−origin *(No. of patients = 61)*

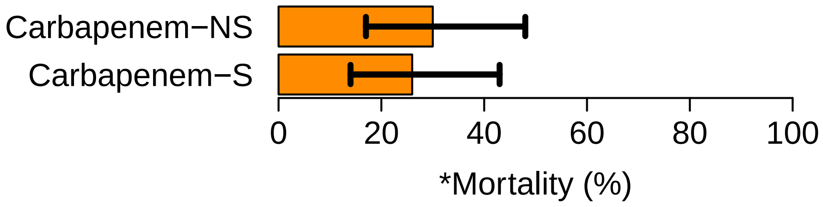

Type of pathogen	Mortality (n)	95% CI
Carbapenem−NS	30% (9/30)	17%−48%
Carbapenem−S	26% (8/31)	14%−43%

Acinetobacter spp. — Hospital−origin *(No. of patients = 172)*

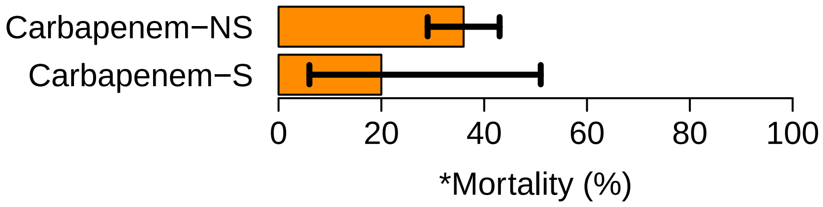

Type of pathogen	Mortality (n)	95% CI
Carbapenem−NS	36% (58/162)	29%−43%
Carbapenem−S	20% (2/10)	6%−51%

*Mortality is the proportion (%) of in−hospital deaths (all−cause deaths). This represents the number of in−hospital deaths (numerator) over the total number of patients with blood culture positive for the organism and the type of pathogen (denominator). The AMASS application de−duplicates the data by including only the first isolate per patient per specimen type per evaluation period.
NS = non−susceptible; S = susceptible; CI = confidence interval; Carbapenem−NS = NS to any carbapenems tested;
3GC−NS = NS to any 3rd−generation cephalosporin and susceptible to carbapenem

Methods used by the AMASS application

Data source:
For each run (double−click on AMASS.bat file), the AMASS application used the microbiology data file (microbiology_data) and the hospital admission data file (hospital_admission_data) that were stored in the same folder as the application file. Hence, if the user would like to update, correct, revise or change the data, the data files in the folder should be updated before the AMASS.bat file is double−clicked again. A new report based on the updated data would then be generated.

Requirements:
− Computer with Microsoft Windows 7 or 10
AMASS may work in other versions of Microsoft Windows and other operating systems. However, thorough testing and adjustment have not been performed.
− AMASS.zip package file
The AMASS application is to be downloaded from http://www.amass.website, and unzipped to generate an AMASS folder that could be stored under any folder in the computer. The AMASS folder contains 4 files (AMASS.bat, z_Rcode.R, dictionary_for_microbiology_data.xlsx, and dictionary_for_hospital_admission_data.xlsx), and 5 folders (Variables, Rprogram, Example_Dataset_1_WHONET, Example_Dataset_2, and ResultData).
− Microbiology data file (microbiology_data in .csv or .xlsx file format)
The user needs to obtain microbiology data, and then copy & paste this data file into the same folder as the AMASS.bat file.
− [Optional] Hospital admission data file (hospital_admission_data)
If available, the user could obtain hospital admission data, and then copy & paste this data file into the same folder as the AMASS.bat file.

Not required:
− Internet to run AMASS application
The AMASS application will run offline. No data are transferred while the application is running and reports are being generated; the reports are in PDF format (do not contain any patient identifier) and can be shared under the user's jurisdiction.
− R
The download package (AMASS.zip) included R portable and R libraries that the AMASS application requires. The user does not need to install any programme before using the AMASS. The user also does not have to uninstall R prgramme if the computer already has the R prgramme installed. The user does not need to know how to use R prgramme.

Note:
[1] Please ensure that the file names of microbiology data file (microbiology_data) and the hospital admission data file (hospital_admission_data) are identical to what is written here. Please make sure that all are lower−cases with an underscore '_' at each space.
[2] Please ensure that both microbiology and hospital admission data files have no empty rows before the row of the variable names (i.e. the variable names are the first row in both files).
[3] For the first run, an user may need to fill the data dictionary files to make sure that the AMASS application understands your variable names and values.

AMASS uses a tier−based approach. In cases when only the microbiology data file with the results of culture positive samples is available, only section one and two would be generated for users. Section three would be generated only when data on admission date are available. This is because these data are required for the stratification by origin of infection. Section four would be generated only when data of specimens with culture negative (no microbial growth) are available in the microbiology data. This is because these are required for the sample−based approach. Section five would be generated only when both data of specimens with culture negative and admission date are available. Section six would be generated only when mortality data are available.

Mortality was calculated from the number of in−hospital deaths (numerator) over the total number of patients with blood culture positive for the organism (denominator). Please note that this is the all−cause mortality calculated using the outcome data in the data file, and may not necessarily represent the mortality directly due to the infections.

How to use data dictionary files
In cases when variable names in the microbiology and hospital admission data files were not the same as the one that AMASS used, the data dictionary files could be edited.
The raw microbiology and hospital admission data files were to be left unchanged.
The data dictionary files provided could be edited and re−used automatically when the microbiology and hospital admission data files were updated and the AMASS.bat were to be double−clicked again (i.e. the data dictionary files would allow the user to re−analyze data files without the need to adjust variable names and data value again every time).

For example:
If variable name for 'hospital number' is written as 'hn' in the raw data file, the user would need to add 'hn' in the cell next to 'hospital_number'. If data value for blood specimens is defined by 'Blood−Hemoculture' in the raw data file, then the user would need to add 'Blood−Hemoculture' in the cell next to 'blood_specimen'.

Dictionary file (dictionary_for_microbiology_data.xlsx) may show up as in the table below:

**Variable names used in AMASS**	**Variable names used in your microbiology data file**	**Requirements**
Don't change values in this column, but you can add rows with similar values if you need	Change values in this column to represent how variable names are written in your raw microbiology data file	
hospital_number		Required
**Values described in AMASS**	**Values used in your microbiology data file**	**Requirements**
blood_specimen		Required

Please fill in your variable names as follows:

**Variable names used in AMASS**	**Variable names used in your microbiology data file**	**Requirements**
Don't change values in this column, but you can add rows with similar values if you need	Change values in this column to represent how variable names are written in your raw microbiology data file	
hospital_number	hn	Required
**Values described in AMASS**	**Values used in your microbiology data file**	**Requirements**
blood_specimen	Blood−Hemoculture	Required

Then, save the file. For every time the user double−clicked AMASS.bat, the application would know that the variable named 'hn' is similar to 'hospital_number' and represents the patient identifier in the analysis.

Organisms included in this report:

- − *Staphylococcus aureus*
- − *Enterococcus* spp.
- − *Streptococcus pneumoniae*
- − *Salmonella* spp.
- − *Escherichia coli*
- − *Klebsiella pneumoniae*
- − *Pseudomonas aeruginosa*
- − *Acinetobacter* spp.

The eight organisms and antibiotics included in the report were selected based on the global priority list of antibiotic resistant bacteria and Global Antimicrobial Resistance Surveillance System (GLASS) of WHO [1,2].

Definitions:

The definitions of infection origin proposed by the WHO GLASS was used [1]. In brief, community−origin bloodstream infection (BSI) was defined for patients in the hospital within the first two calendar days of admission when the first blood culture positive specimens were taken. Hospital−origin BSI was defined for patients in the hospital longer than the first calendar days of admission when the first blood culture positive specimens were taken. In cases when the user had additional data on infection origin defined by infection control team or based on referral data, the user could edit the data dictionary file (variable name 'infection_origin') and the AMASS application would use the data of that variable to stratify the data by origin of infection instead of the above definition. However, in cases when data on infection origin were not available (as in many hospitals in LMICs), the above definition would be calculated based on admission date and specimen collection date (with cutoff of 2 calendar days) and used to classify infections as community−origin or hospital−origin.

De−duplication:

When more than one blood culture was collected during patient management, duplicated findings of the same patient were excluded (de−duplicated). Only one result was reported for each patient per sample type (blood) and surveyed organisms (listed above). For example, if two blood cultures from the same patient had *E. coli*, only the first would be included in the report. If there was growth of *E. coli* in one blood culture and of *K. pneumoniae* in the other blood culture, then both results would be reported. One would be for the report on *E. coli* and the other one would be for the report on *K. pneumoniae.*

References:

[1] World Health Organization (2018) Global Antimicrobial Resistance Surveillance System (GLASS) Report. Early implantation 2016−2017. http://apps.who.int/iris/bitstream/handle/10665/259744/9789241513449−eng.pdf. (accessed on 3 Dec 2018)

[2] World Health Organization (2017) Global priority list of antibiotic−resistant bacteria to guide research, discovery, and development of new antibiotics. https://www.who.int/medicines/publications/WHO−PPL−Short_Summary_25Feb−ET_NM_WHO.pdf. (accessed on 3 Dec 2018)

Investigator team

The AMASS application is being developed by Cherry Lim, Clare Ling, Elizabeth Ashley, Paul Turner, Rahul Batra, Rogier van Doorn, Soawapak Hinjoy, Sopon Iamsirithaworn, Susanna Dunachie, Tri Wangrangsimakul, Viriya Hantrakun, William Schilling, John Stelling, Jonathan Edgeworth, Guy Thwaites, Nicholas PJ Day, Ben Cooper and Direk Limmathurotskul.

The AMASS application was funded by the Wellcome Trust (grant no. 206736 and 101103). C.L. is funded by a Training Research Fellowship (grant no. 206736) and D.L. is funded by an Intermediate Training Fellowship (grant no. 101103) from the Wellcome Trust.
