# Supplementary material for: Automating the Generation of Antimicrobial Resistance Surveillance Reports: Proof-of-Concept Study Involving Seven Hospitals in Seven Countries
Source: J Med Internet Res. 2020 Oct 2;22(10):e19762. doi: 10.2196/19762 (PMC7568216; doi:10.2196/19762)
Supplement: Multimedia Appendix 9 [file jmir_v22i10e19762_app9.docx]

**Multimedia Appendix 7:** The list of participating hospitals.

| **Hospital** | **City** | **Country** | **Availability of AST result data*** | **Availability of negative culture data** | **Availability of hospital admission date data** | **Availability of outcome data**** | **Sections of AMR report that are available** | **Website** |
| --- | --- | --- | --- | --- | --- | --- | --- | --- |
| Angkor Hospital for Children | Siem Reap | Cambodia | Yes | Yes | Yes | Yes | Section 1, 2, 3, 4, 5 and 6 | <https://doi.org/10.6084/m9.figshare.12000225.v1> |
| Mahosot Hospital | Vientiane | Lao PDR | Yes | No | No | No | Section 1 and 2 | <https://doi.org/10.6084/m9.figshare.12000222.v1> |
| North Okkalapa General and Teaching Hospital | Yangon | Myanmar | Yes | Yes | Yes | Yes | Section 1, 2, 3, 4, 5 and 6 | <https://doi.org/10.6084/m9.figshare.12000237.v1> |
| Patan Hospital | Kathmandu | Nepal | Yes | No | No | No | Section 1 and 2 | <https://doi.org/10.6084/m9.figshare.12000231.v1> |
| Sunpasitthiprasong Hospital | Ubon Ratchathani | Thailand | Yes | Yes | Yes | Yes | Section 1, 2, 3, 4, 5 and 6 | <https://doi.org/10.6084/m9.figshare.12000240.v1> |
| St Thomas' Hospital | London | the United Kingdom | Yes | No | Yes | Yes | Section 1, 2, 3 and 6 | <https://doi.org/10.6084/m9.figshare.12000249.v1> |
| Hospital for Tropical Diseases | Ho Chi Minh City | Vietnam | Yes | Yes | No | No | Section 1, 2 and 4 | <https://doi.org/10.6084/m9.figshare.12000252.v1> |

Footnote of Supplementary Table 2. *Antimicrobial susceptibility testing (AST) data on interpretative categories of susceptible, intermediate, and resistant. **In-hospital discharge outcome.
