# Supplementary material for: Automating the Generation of Antimicrobial Resistance Surveillance Reports: Proof-of-Concept Study Involving Seven Hospitals in Seven Countries
Source: J Med Internet Res. 2020 Oct 2;22(10):e19762. doi: 10.2196/19762 (PMC7568216; doi:10.2196/19762)
Supplement: Multimedia Appendix 10 [file jmir_v22i10e19762_app10.docx]

**Multimedia Appendix 8:** How to export data from WHONET 5.6 (2019) in an Excel format that can be used by AMASS. The process helps users with raw disk diffusion zone diameter/MIC values to convert to antimicrobial susceptibility test interpretive categories (susceptible, intermediate, resistant). Data analysis menu (A, C, E, G and I) brings users to separated windows (B, D, F and H), which contains various functions to process the data.

**Footnote of Supplementary Figure 2.** The WHONET users may follow the steps below to combine several data files into one, and to standardardise the format to export in Excel format for AMASS. Please note that we have created a data dictionary that is downloadable from [www.amass.website](http://www.amass.website) for the WHONET users.

1) Select “Data analysis” under the “Data analysis” tab.

2) Click “Analysis type” button on the “Data analysis” window.

3) Select “Isolate listing and summary” tab.

4) Click “1. Listing” option.

5) Click “OK” to complete and exit the window.

6) Click “Options” button on the “Data analysis” window.

7) Select “Test interpretations” option under “Isolate listing and summary”

8) Click “OK” to complete and exit the window.

9) Click “Organisms” button on the “Data analysis” window.

10) Select ALL by highlighting the list or type “ALL” in the blank space next to “Code”.

11) Click the right arrow to include the whole list.

12) Click “OK” to complete and exit the window.

13) Click “Data files” button on the “Data analysis” window.

14) Select the data set(s) to be exported.

15) Click the right arrow to include the data set(s) to be exported.

16) Click “OK” to complete and exit the window.

17) Choose “Excel” in the drop-down menu of the “Output to:”.

18) Name the file to be exported.

19) Click “Begin analysis” to export the data.
